# Genomic changes associated with adaptation to arid environments in cactophilic *Drosophila* species

**DOI:** 10.1186/s12864-018-5413-3

**Published:** 2019-01-16

**Authors:** Rahul V. Rane, Stephen L. Pearce, Fang Li, Chris Coppin, Michele Schiffer, Jennifer Shirriffs, Carla M. Sgrò, Philippa C. Griffin, Goujie Zhang, Siu F. Lee, Ary A. Hoffmann, John G. Oakeshott

**Affiliations:** 1 CSIRO, Clunies Ross St, GPO Box 1700, Acton, ACT 2601 Australia; 2 Bio21 Institute, School of BioSciences, University of Melbourne, 30 Flemington Road, Parkville, 3010 Australia; 3 China National GeneBank, BGI-Shenzhen, Shenzhen, China; 4 School of Biological Sciences, Monash University, Melbourne, 3800 Australia; 5 Centre for Social Evolution, Department of Biology, University of Copenhagen, Universitetsparken 15, København, Denmark

**Keywords:** Comparative genomics, Transcriptomics, Cactophilic Drosophila, Heat stress, Host adaptation

## Abstract

**Background:**

Insights into the genetic capacities of species to adapt to future climate change can be gained by using comparative genomic and transcriptomic data to reconstruct the genetic changes associated with such adaptations in the past. Here we investigate the genetic changes associated with adaptation to arid environments, specifically climatic extremes and new cactus hosts, through such an analysis of five *repleta* group *Drosophila* species.

**Results:**

We find disproportionately high rates of gene gains in internal branches in the species’ phylogeny where cactus use and subsequently cactus specialisation and high heat and desiccation tolerance evolved. The terminal branch leading to the most heat and desiccation resistant species, *Drosophila aldrichi*, also shows disproportionately high rates of both gene gains and positive selection. Several Gene Ontology terms related to metabolism were enriched in gene gain events in lineages where cactus use was evolving, while some regulatory and developmental genes were strongly selected in the *Drosophila aldrichi* branch. Transcriptomic analysis of flies subjected to sublethal heat shocks showed many more downregulation responses to the stress in a heat sensitive versus heat resistant species, confirming the existence of widespread regulatory as well as structural changes in the species’ differing adaptations. Gene Ontology terms related to metabolism were enriched in the differentially expressed genes in the resistant species while terms related to stress response were over-represented in the sensitive one.

**Conclusion:**

Adaptations to new cactus hosts and hot desiccating environments were associated with periods of accelerated evolutionary change in diverse biochemistries. The hundreds of genes involved suggest adaptations of this sort would be difficult to achieve in the timeframes projected for anthropogenic climate change.

**Electronic supplementary material:**

The online version of this article (10.1186/s12864-018-5413-3) contains supplementary material, which is available to authorized users.

## Background

One approach to assessing the ability of species to adapt genetically to future climate change is to reconstruct the way such adaptation has occurred in the past. The best way to do this is to compare the genomes of closely related species that have diverged for the relevant phenotypes, but where genetic changes due to drift or other adaptations irrelevant to those phenotypes are minimal [1]. *Drosophila* is an ideal genus for such an analysis because many of its species have diverged in their responses to climatic extremes [2–4].

One particularly promising species group to study in this respect is the *repleta* group (subgenus *Drosophila*), which originated about 15 million years ago in the Americas [5]. Many species in this group, such as the *mulleri* subgroup species *Drosophila mojavensis*, *D. buzzatii* and *D. aldrichi*, are desert-adapted and display extremely high heat, cold and desiccation tolerance [2, 3] but other species, such as the *hydei* and *repleta* subgroup species *D. hydei* and *D. repleta*, are largely found outside the desert and are much less tolerant of these stresses [2, 3, 6]. Notably also, while all the *repleta* group species are saprophagous (feed on rotting tissue) they vary widely in their host preferences; the desert species are dietary specialists that feed and breed on necrotic cactus tissue, whereas *D. hydei* and *D. repleta* are dietary generalists which can utilise a wide range of rotting fruits and vegetables, as well as animal faeces and, in the case of *D. hydei*, cacti as well [7–10].

Some comparative genomic studies have been conducted on two *repleta* group species, the cactophilic *D. mojavensis* and *D. buzzatii*. Both are relatively tolerant to climate stresses [5, 11, 12] but the former is much more restricted geographically and in the range of cacti it will use [13]. Comparisons between these two species and two other drosophilids outside the *repleta* species group (*D. virilis* and *D. grimshawi*) showed expansions of gene families involved in proteolysis, sensory perception and gene regulation in the cactophilic species [5]. The same study also found the cactophilic species were undergoing rapid positive selection in genes involved in gene regulation and the catabolism of some of the heterocyclic toxins found in the cacti [5]. Transcriptomic comparisons of populations living on different hosts within both *D. mojavensis* and another *repleta* species group cactophile, *D. mettleri*, have also highlighted transcriptional changes in key metabolic and sensory pathways which might contribute to desiccation and/or host adaptation [12, 14–16]. There is thus evidence for both regulatory and structural changes, in the form of gene gains as well as positive selection, associated with the acquisition of cactophilism in the *repleta* group. However interpretation of the associations is limited by the few species studied and in some cases the substantial phylogenetic distance involved in the comparison.

Several genome-wide association (GWAS) studies have also found quantitative trait loci (QTLs) contributing to polymorphic variation in thermal and desiccation stress traits within *D. melanogaster* [17–20]. Associations have been recorded with hundreds of different genes, including a number of heat shock proteins, but their relevance to the cactophilic *repleta* species is questionable because of the ecological differences and phylogenetic distance involved, and the fact that most of the *D. melanogaster* studies are based on microarray rather than sequencing data.

To follow up the work on the cactophilic species above, the current study investigates gene gains and positive selection in five sequenced *repleta* group species, and transcriptional differences in two of them with very different thermal tolerances. The five species are *D. mojavensis* and *D. buzzatii*, plus one additional highly stress tolerant cactophile, *D. aldrichi* (specifically its Clade A; [21]), and two less tolerant dietary generalists, *D. repleta* and *D. hydei*. We use the *D. mojavensis* genome (generated from its Catalina Island clade; [22]) as published but we re-annotate the published *D. buzzatii* genome [5] to improve gene model prediction for that species. We present new genomes for the other three species, acknowledging that another version of the *D. hydei* genome has also recently been published ([23] and see below). Comparative analyses among these four genomes plus *D. mojavensis*, and between the five *repleta* species and previously published genomes from other *Drosophila* groups, are then used to suggest genetic factors contributing to high temperature tolerance and cactus vs generalist dietary adaptations. These analyses are founded on a robust genome-wide phylogeny for a total of 24 *Drosophila* species for which good quality genomes were available at the time [24]. Orthologue and duplication predictions and branch site modelling are then used to identify lineage-specific gene expansions and bursts of positive selection in the *repleta* species group. We also compare transcriptomes across a time course of heat shock response for the heat sensitive *D. hydei* and heat tolerant *D. buzzatii*, and test whether gene sets showing divergent transcriptomic responses to heat between these species are related to those showing genomic divergence.

## Results

### Genome assemblies and annotations

Among the three newly sequenced species, the highly inbred *D. hydei* and *D. repleta* lines had better assembly statistics than the *D. aldrichi* line, which was less inbred than the other two (see Materials and Methods and Additional file 1: Text S2). This is apparent from the larger scaffolds and smaller scaffold L50 s for *D. hydei* and *D. repleta* compared to *D. aldrichi* (Additional file 2: Table S1). The *D. hydei* assembly also had superior assembly (and annotation) statistics to the other recently published version of this genome [23]; compared to the other version, our assembly had an N50 three times larger and covered 90% of the genome in less than half the number of scaffolds (Additional file 2: Tables S1, S2).

The generalist feeders *D. hydei* and *D. repleta* yielded assembled genome sizes of ~ 165 Mb, which is very close to previous estimates generated using DAPI staining (177+/− 22 and 167+/− 13 Mb respectively; [25]). No DAPI estimate has been published for the cactophilic *D. aldrichi* but our assembled genome size (191 Mb) for this species was larger than the two generalists but similar to that previously published for the cactophilic *D. mojavensis* (194 Mb; [26]), which itself was corroborated by a DAPI estimate (183+/− 3 Mb; [25]). Notably the previously published assembly of the other cactophilic species in our analysis, *D. buzzatii*, was only estimated at 161 Mb, most likely due to significant underestimation of both repeat and gene content during genome assembly ([27] and see below).

The repeat contents of the various genomes were highly variable across the five assemblies (Additional file 2: Table S3). At the extremes, *D. buzzatii* had only 8.4% total repeat content while *D. aldrichi* had 24.5%*.* The lower *D. buzzatii* estimate however may in part reflect the tendency of strict short read deBruijn graph assemblies such as that one to underestimate repeat contents [27].

We identified 15,838, 14,790 and 16,070 genes respectively in *D. hydei*, *D. repleta* and *D. aldrichi*, most of which have annotated UTRs (Additional file 2: Table S3). Our gene numbers for these three species are slightly higher than the 14,680 and 13,919 published for *D. mojavensis* and *D. melanogaster* respectively, and higher again than the 13,158 published for *D. buzzatii*. We think this is because the annotation pipeline used in our study yields superior recovery and quality statistics than many of its predecessors [24]. A reannotation of the published *D. buzzatii* data resulted in the identification of 1374 additional genes (Additional file 2: Table S3) and brings the total gene numbers up to 14,532, which is at the lower end of the range found for the other *repleta* group species. The new annotation also increased the number of identifiable orthologous genes in *D. buzzatii* by 485 (Additional file 2: Table S4). Scans for members of the conserved CEGMA [28] and BUSCO [29] gene sets suggested that our new *D. buzzatii* annotation was still missing about 10% of genes, compared with less than 3% for the other species (data not shown). Accounting for these missing genes would leave *D. buzzatii* with similar gene numbers to *D. hydei*, suggesting no consistent difference in gene number between the cactophilic and non-cactophilic species.

Orthonome [24] was used to identify orthologues, inparalogues (species-specific duplications) and de novo gene births (of which there were very few) in the gene sets of these and 11 other *Drosophila* species (see Materials and Methods). Orthonome classifies sequences as orthologues if they meet three criteria relating to sequence similarity, synteny and, where duplications have occurred, symmetric sister group phylogenetic relationships (see Materials and Methods); sequences only meeting one or two of these criteria were classified as inparalogues and those meeting none classified as gene births. Orthonome and Interproscan (v5.16–55; [30]) were also used to place 87–91% of the genes in each species in various Gene Ontology (GO) categories [31].

### Phylogenomics shows a progression from host generalists to cactophilic specialists

**Fig. 1:**
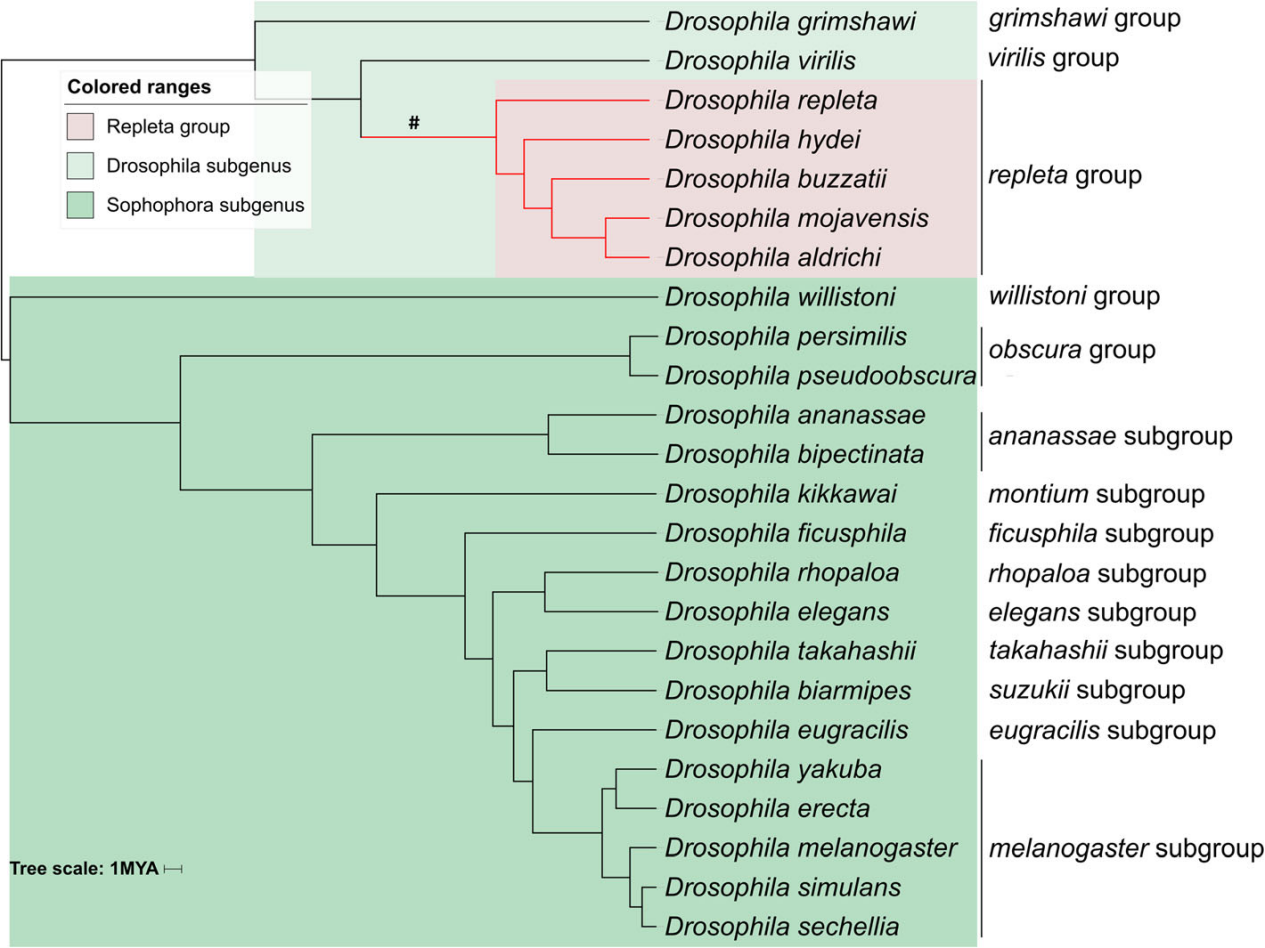
Phylogenetic relationships of the five *repleta* group species and 19 previously sequenced Drosophila species based on concatenated codon alignments of 1802 orthogroups shared by all species. The divergence time was estimated using the RelTime [102] package in MEGA7 [93]. All bootstrap values for nodes from 1000 iterations were equal to 1 and the ancestral *repleta* group branch is indicated with a hash

IQ-Tree [32] was used to obtain a species phylogeny from the nucleotide coding sequences of 1802 orthogroups (groups of 1:1 orthologues containing no inparalogues) that had members in all five *repleta* group species plus the 19 other *Drosophila* species for which the Drosophila 12 genomes (ftp://ftp.flybase.net/genomes/) and modENCODE (https://www.hgsc.bcm.edu/arthropods/drosophila-modencode-project) projects produced good quality genome sequences. The well-resolved tree obtained (100% bootstrap support for all branches in Fig. 1) generally confirmed earlier phylogenies for the *repleta* group [5, 13, 33]. Specifically it partitioned the cactophilic species, which are all in the *mulleri* subgroup, into the *mulleri* complex species *D. aldrichi* and *D. mojavensis* and the *buzzatii* complex species *D. buzzatii*, with *D. hydei* (in the *hydei* subgroup) the sister clade to that subgroup and *D. repleta* (in the *repleta* subgroup) the sister for all four of the other species.

Notably in the latter respect our phylogeny agreed with one, but not the other, of the only two previously published phylogenies we know of which included both *D. hydei* and *D. repleta.* One of these, by ourselves, which was based on similar methods as here and 257 genes from a partially overlapping set of species, also put the *D. repleta* split ancestral to that of *D. hydei* [34]. The other, by Oliviera et al. [13], which applied maximum likelihood methods to four mitochondrial and six nuclear gene fragments, placed *D. hydei* as the outgroup to the other four species and in so doing suggested *D. repleta* had lost an ability to use cactus that had itself emerged in the common ancestor of it and *D. hydei*. In putting *D. repleta* as an outgroup to the cactus-using species, our current and earlier study do not require *D. repleta* to have secondarily lost that ability. *D. hydei*’s ability to eat both fruits/vegetables and faeces as per *D. repleta* and cacti as per the *mulleri* subgroup is also consistent with its positioning in our topology between the *D. repleta* and *mulleri* subgroup branches. (We note here that the *nannoptera* species group and its most closely related outgroup *D. machalilla*, which all also sit in the large *virilis-repleta* radiation, have independently evolved cactophilism, in their case apparently from flower-feeding ancestors [5, 35].)

We also note that our phylogenetic methods were primarily built upon the supermatrix approach used in the i5K study [36] and only used carefully selected orthologues to run ModelFinder and IQ-Tree ([32, 37] and see Methods below). We obtained the same topology if we used even more stringent filtering of input genes (to remove 22% of the genes which showed compositional heterogeneity [38]) or another concatenated sequence approach using FastTree2 [39] (analyses not shown). On the other hand, we found that a Bayesian consensus tree approach using *BEAST2 [40] could not separate the two divergences and gave lower concordance values [41] and partitioned coalescence support (PCS) [42] across the gene set used (data not shown). We conclude that the two divergences in question are likely very close to one another but the topology with *D. repleta* as the outgroup which we propose has stronger statistical support.

Our dating analysis does however agree with Oliviera et al. [13] in suggesting an origin for the *repleta* group around 10–15 MYA (Fig. 1). This was a period of aridification through parts of the Americas where the group is believed to have evolved, together with the flat-leaved Opuntioideae believed to be the hosts for the original cactophiles within the group [13].

Our genome-based tree also clarified some relationships in the *Sophophoran* subgenus, as detailed in the Additional file 1: Text S1.

### Orthogroup generation rate is high when cactophilism evolves but low in the specialist *mulleri* complex branch

**Fig. 2:**
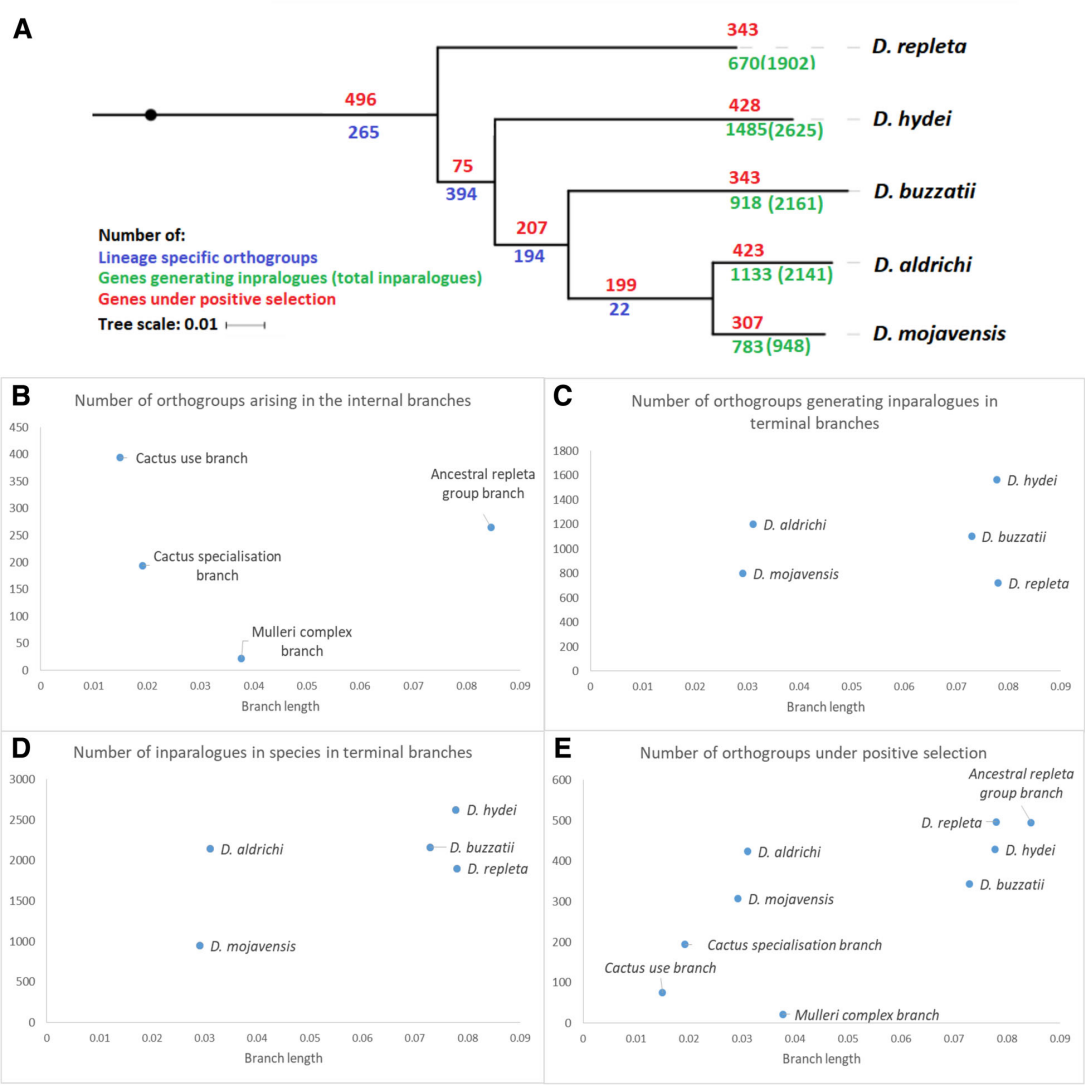
Consensus phylogenetic tree estimated using IQ-Tree with 1802 genes and 1000 bootstraps for the *repleta* groups species showing the numbers of orthogoups arising in the internal branches ((blue) and generating inparalogues in the terminal branches (green), plus the total numbers of inparalogues in the terminal branches (brackets, green) and the number of positively selected genes in all branches (red), togther with plots of these four metrics against the lengths of the respective branches

Application of Orthonome to the five *repleta* species and the 11 other species in the Drosophila 12 genomes project (the somewhat lesser quality modENCODE genomes being excluded from this analysis) identified orthologues for 11,780–13,883 genes in each of the 16 species (Table 1, Additional file 2: Table S4). These orthologues were distributed across 15,907 orthogroups, 971 of which orthogroups arose in the four internal branches within the *repleta* group phylogeny. Figure 2 shows that 265 of the 971 orthogroups arose in the earliest of these branches (hereafter the ancestral *repleta* group branch), 394 in the next one (ie after the split of the four cactus using species off from *D. repleta*, hereafter the cactus use branch), 194 in the next (ie following the split of the three cactus specialists off from *D. hydei*, hereafter the cactus specialisation branch), and just 22 in the most recent one (ie after the two *mulleri* complex species split off from *D. buzzatii*, hereafter the *mulleri* complex branch). Thus, in proportion to the presumptively neutral silent site branch lengths (calculated from 4-fold degenerate sites in 1802 full length genes as detailed in the Methods section, the rate of orthogroup creation was highest for the branches in which cactus feeding and then, to a lesser extent, cactophilic specialisation evolved, and lowest in the *mulleri* complex branch within the cactophilic specialists (Fig. 2). The excess of orthogroups arising in the cactus use and cactus specialisation branches in proportion to their silent site branch lengths was of the order of 300 and 150 orthogroups respectively. Relevant here is that the cactus specialisation branch is not only associated with the substantive loss of use of other hosts but also the acquisition of greater heat and desiccation tolerance, all three of the cactophilic species having significantly higher tolerance than *D. repleta* and *D. hydei* [2, 3, 43–47].

**Table 1:** Summary of Orthonome analysis for *D. melanogaster* and five *repleta* group species. Average numbers of one-to-one orthologues (genes identified by Orthonome as having at least one orthologous relationship) are calculated based on pairwise orthologue predictions whereas total genes with orthologues and inparalogues are evaluated using the phylogeny-sensitive Orthonome analysis of all genomes

Species	Number of genes	Mean number of 1:1 orthologues	Total genes with orthologues	Inparalogues	
				*All orthogroups*	*Repleta group-specific orthogroups*
*D. aldrichi*	16,070	11,294	12,469	2141	100
*D. mojavensis*	14,680	11,669	13,147	948	69
*D. buzzatii* (improvement compared to original annotation)	14,532 (1374)	10,745 (485)	11,780	2161	173
*D. hydei*	15,838	11,096	12,523	2625	514
*D. repleta*	14,790	11,222	12,588	1902	224
*D. melanogaster*	13,919	10,894	12,992	688	NA

In fact the rate estimated for the cactus specialisation branch may have been slightly underestimated, and that for the *mulleri* complex slightly overestimated, because of the slightly lower rate of gene recovery from our *D. buzzatii* assembly noted above (which would have meant we assigned a few orthogroups which actually arose in the cactus specialisation branch to the *mulleri* complex branch).

### Inparalogue generation rates are high in *D. aldrichi*

Orthonome also identified 948 (*D. mojavensis*) - 2625 (*D. hydei*) inparalogues arising in the terminal branches of the *repleta* group phylogeny, with the number of orthogroups giving rise to them varying between 783 (*D. mojavensis*) and 1485 (*D. hydei*) (Table 1; Fig. 2). Much of this variation was broadly proportional to the silent site branch lengths. However the most heat and desiccation resistant species, *D. aldrichi* [2, 3, 43–47], had about twice as many inparalogues and orthogroups giving rise to them (excesses of several hundred in both cases) as might be expected from its silent site branch length. The high number of inparalogues in this species was not obviously explicable in terms of its slightly poorer quality assembly (see above); only 171 out of 2140 of its inparalogues were found at the same locations as their templates in the assembly and were therefore possibly assembly errors (data not shown). On the other hand, inparalogue numbers in *D. buzzatii* may have been slightly underestimated because of the slightly lower rate of gene recovery from its assembly.

Overall, the terminal branch inparalogue numbers estimated are significantly higher in proportion to the silent site branch lengths than are the numbers of orthogroups which were estimated above to have been generated in the internal branches. This may in part be a statistical artefact; the very rigorous criteria we used for the classification of orthogroups, may mean some real orthologues were assigned as inparalogues (see Materials and Methods). However it likely also has some biological basis; only duplication events that survive long enough as functioning genes to appear in at least two of the five species will be classified as orthogroups, whereas the inparalogues will include some functionless genes, not necessarily transcribed and possibly including disruptions to open reading frames.

There was no obvious correlation between the specific orthogroups generating inparalogues in the different terminal branches (Additional file 2: Figure S1). This would indicate ongoing genetic divergence among the species, even among the cactophiles. As noted, there are considerable differences in the heat and desiccation tolerances of the five species and even the cactophiles differ in host use to a significant degree, specifically in their preferences for various flat-leaved Opuntia species (*D. buzzatii*) versus specific columnar cacti (the two *mulleri* complex species, particularly *D. mojavensis* [33]).

### Many positively selected genes in *D. aldrichi* but few in the internal *mulleri* complex branch

Adaptive branch-site random effects likelihood (aBSREL; [48]) testing of 7359 orthogroups with 1:1 orthologues in each of the five *repleta* species and the 11 other FlyBase species found between 75 and 496 orthogroups undergoing positive selection in one or more branches of the *repleta* group phylogeny (Fig. 2). In proportion to the respective silent site branch lengths, the major outliers were the terminal branch containing *D. aldrichi*, which showed relatively high numbers of positively selected genes, and the preceding internal branch, the *mulleri* complex branch, which contained relatively few (of the order of 200 more and 200 less respectively). *Drosophila aldrichi*, which has the highest heat and desiccation tolerance of the species characterised [2, 3, 43–47], was also associated with relatively high numbers of gene gains (as assessed by inparalogues generated), consistent with its ecological niche having diverged significantly from the others species. Conversely, the *mulleri* complex branch showed very low relative numbers of both gene gain events and positively selected genes, suggesting a period of relative evolutionary genetic stability. As with the inparalogue analyses, there was little overall correlation between the specific genes showing positive selection in the different branches, even among the terminal cactus specialist branches (Additional file 2: Figure S2).

We note here that this analysis only included the 7359 orthogroups/genes which had orthologues in all 16 species analysed. While this was the most rigorous way to execute the test, it also meant that many orthogroups which were more variable in terms of their presence across species were excluded from the analysis. The analyses below suggest that orthogroups associated with gene gains are more likely to show positive selection than those that do not. These considerations suggest that our estimates of the proportions of genes under positive selection in the various branches above will underestimate the true proportions to some degree.

### Genes generating inparalogues are more likely to be positively selected

We used the approach developed by O’Toole et al. [49] to test whether there was an association between the orthogroups/genes generating inparalogues and those that were under positive selection. The structure of this analysis necessarily restricted it to the terminal branches of the phylogeny but a fortunate corollary of this was that we could include more genes than in the aBSREL analysis for positive selection above (see Materials and Methods); specifically we could include all orthogroups which had members in all the *repleta* group species and at least one other species from the same subgenus. We found that genes generating inparalogues in the terminal branches were more likely to be under positive selection than genes which had not generated inparalogues in those branches (Table 2), although the analysis does not tell us whether the episodes of positive selection involved occurred before or after the generation of the inparalogues.

**Table 2:** Proportions of genes with or without inparalogues that are under positive selection. 95% binomial confidence intervals are shown for the percentages in parentheses

Species	% positively selected genes lacking inparalogues	% positively selected genes generating inparalogues
*D. aldrichi*	429/9615 = 4.47 (0.41, 0.49)	95/1237 = 7.68 (6.26, 9.31)
*D. mojavensis*	348/10740 = 3.25 (0.29, 0.36)	21/431 = 4.88 (3.04, 7.35)
*D. buzzatii*	327/8951 = 3.66 (0.33, 0.41)	86/1173 = 7.34 (5.91, 8.98)
*D. hydei*	496/10070 = 4.93 (0.45, 0.54)	36/692 = 5.21 (3.67, 7.13)
*D. repleta*	371/10480 = 3.55 (0.32, 0.39)	31/456 = 6.80 (4.67, 9.51)

### Metabolic and stress response functions are enriched in the evolutionary changes

**Fig. 3:**
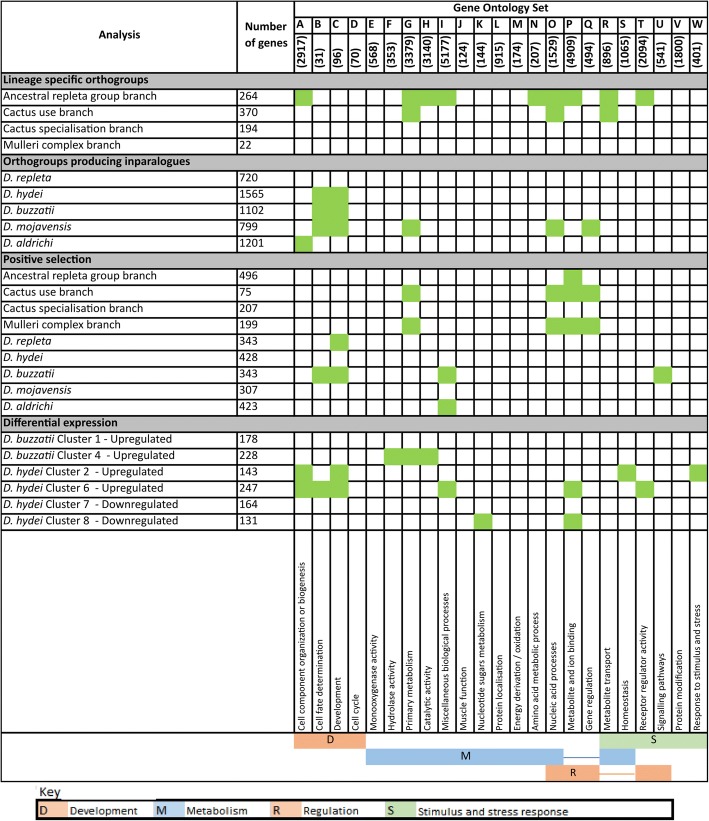
Summary of significant enrichments (FDR-corrected *P* < 0.05) of 23 sets of GO terms for biological processes in the 18 evolutionary analyses in Fig. 2 and in the over-represented species in the six key fuzzy-c means clusters from the heat stress transcriptome analyses (Fig. 5 below)

We screened the 18 branch-specific evolutionary analyses above (orthogroups created in the four internal branches, orthogroups generating inparalogues in the five terminal branches and positive selection in each of the nine branches) for the enrichment of 23 mutually exclusive sets of GO terms. These terms were obtained by applying a Louvain clustering approach to all 28,912 terms nested under ‘Biological process’ in the Ontology (see Materials and Methods). We then examined the associations found in more detail by analysing the enrichments of 265 subsets of the 23 sets, again identified by Louvain clustering. The constitutions of the different GO sets and subsets are summarised in Additional file 2: Table S5. Twenty eight significant enrichments of GO sets were found and these 28 were not randomly distributed with respect to either the analyses or the GO sets involved (Fig. 3).

Nine of the 28 occurred in just one analysis, namely that assessing orthogroup generation in the ancestral *repleta* group branch. While this branch had not generated disproportionate numbers of orthogroups relative to its silent site branch length, it was nevertheless a relatively long silent site branch. The range of biological functions enriched among the orthogroups generated in this branch may have been prerequisites for the emergence of the very broad host range of *D. repleta* itself, the flower feeding habits of the ancestors of the *nannoptera* group, fungus feeding in some *fasciola* subgroup species, the cactophilic lineage studied here, plus the soil-dwelling *D. mettleri* that feeds on the exudate of rotting cactus [13, 15]. Another 12 of the 28 significant enrichments occurred in just three other analyses, three in orthogroups arising in the cactus use branch (which had shown a pronounced excess of events relative to the silent site branch length), five in orthogroups generating inparalogues in the *D. mojavensis* branch (which involved increasing specialisation on particular chemically complex columnar cacti), and four in positive selection events in the *D. buzzatii* branch (which continues to exploit a wide range of cacti).

Five GO sets were enriched in two or more of the 28 analyses; three (G - Primary metabolism, O - Nucleic acid processes and P - Metabolite and ion transport) in the orthogroups generated in both the ancestral *repleta* group and cactus use branches above, and two others (B - Cell fate determination and C - Development) each involved in four terminal branch analyses, namely the orthogroups generating inparalogues in the *D. hydei*, *D. buzzatii* and *D. mojavensis* branches and positive selection in the *D. buzzatii* branch.

**Fig. 4:**
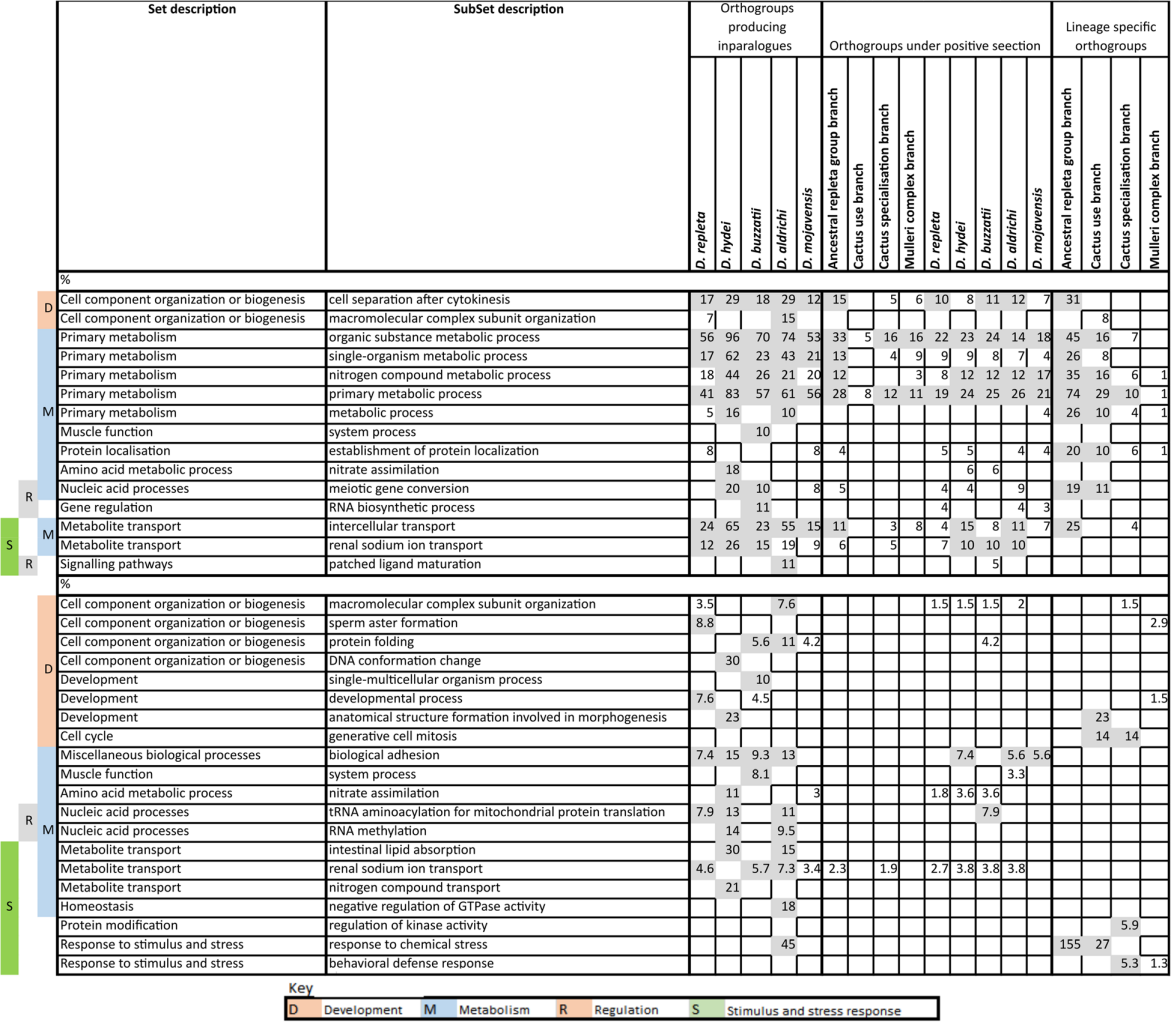
GO subsets most represented in the 18 evolutionary analyses, where representation is expressed in terms of either the total number (top) or percentage (below) of genes in the subset involved in the respective gene gain or positive selection events. Only subsets containing more than ten genes were analysed and only those where more than ten genes (top) or more than 5% (below) were involved in the events in any one of the analyses are shown. These values are shaded. However for those subsets included on these criteria, any non-zero involvement in any of the other analyses is also shown, but not shaded. See the Methods section for further details of the methods used to generate this figure

We then screened all the GO subsets for enrichments in the 18 analyses, which we did in terms of both absolute number and percentage increases (Fig. 4). Consistent with the set-level analyses, increases in absolute numbers were most evident for the orthogroups generated in the ancestral *repleta* group and cactus use branches and the orthogroups generating inparalogues in most of the terminal branches, with less marked increases in the positive selection analyses. Notably the four subsets contributing most strongly to these increases were all subsets of the Primary metabolism set (G), and three of these, Organic substance metabolic processes, Nitrogen compound metabolic processes and Primary metabolic process, would have obvious relevance to host use, and in particular the very different carbon and nitrogen contents of cactus over rotting fruit diets [12].

The percentage increase data were more informative for the smaller subsets. While they generally showed patchier distributions across analyses and GO subsets, they nevertheless did identify some relatively large effects for three subsets of Metabolite and ion transport (P) and two subsets of Response to stimulus and stress (W), which again could relate to the acquisition and specialisation of cactophilism.

### Evolution in the *D. aldrichi* branch involves many functions

We were particularly interested to screen for the biochemical functions implicated in the bursts of inparalogue generation and positive selection in the terminal *D. aldrichi* branch. The GO set-level analysis above showed it was significantly enriched for two relatively ill-defined sets, namely A (Cell component organisation or biogenesis) and I (Miscellaneous biological processes), for inparalogue generation and positive selection respectively. The subset-level analyses also showed this branch was enriched for a wide range of terms, most of which were also involved to some degree in one or more analyses of other branches. These analyses suggest that a broad range of functions were involved in the evolution occurring in this branch, with little differentiation between these genes and those involved in some of the other branches.

To further interrogate the functions involved we then looked at the functional annotations of the 20 genes most strongly implicated (ie having the lowest *P* values) in this branch in the positive selection screen above (Table 3). Most of these 20 genes had regulatory or developmental functions, suggesting such functions might have been particular targets for the positive selection in this branch. Unfortunately we could not do the equivalent analysis for the inparalogues generated in this branch because the nature of those data prohibited any ranking of genes in terms of strength of the effect.

**Table 3:** Top 20 genes undergoing positive selection in *D. aldrichi*. Orthogroups with Bonferroni corrected *p*-value < 1.0e^− 20^ and Likelihood ratio test statistic estimate from aBSREL > 82. Functional information for each gene was summarised based on Flybase descriptions for *D. melanogaster* orthologues, classification of the protein sequence using gene ontology and/or Pfam family annotations. Asterisks indicate two genes which also have inparalogues in the *D. aldrichi* branch

*D. aldrichi* gene	*D. melanogaster* orthologue	Gene name	Gene function
DALD015788*	FBgn0001341	lethal (1) 1Bi (*l(1)1Bi*)	Transcriptional regulator during larval and embryo stages
DALD015521	FBgn0034583	CG10527	DUF3421; Farnesoic acid O-methyl transferase
DALD011516	FBgn0034031	CG12963	NA
DALD011555	FBgn0029518	CG13376	NA
DALD014639	FBgn0038654	CG14298	Serine-type endopeptidase inhibitor activity
DALD008141	FBgn0037244	CG14647	Transcriptional regulator of protein homo-oligomerisation
DALD001586	FBgn0029686	CG2941	NA
DALD015580*	FBgn0050440	CG30440	Involved in positive regulation of Rho protein signal transduction
DALD010188	FBgn0266566	CG45105	Centrosome organising gene
DALD004399	FBgn0038921	CG6332	Testicular haploid expressed repeat
DALD000608	FBgn0036179	CG7368	Transcription factor regulating phagocytosis
DALD007329	FBgn0045495	Gustatory receptor 28b (*Gr28b*)	Taste receptor involved in perception of chemical stimuli
DALD007082	FBgn0032416	Gustatory receptor 33a (*Gr33a*)	Taste receptor involved in perception of chemical stimuli and courtship behaviour
DALD009511	FBgn0031275	metabotropic GABA-B receptor subtype 3 (*GABA-B-R3*)	G-protein coupled receptor regulating glucose metabolic processes
DALD010061	FBgn0051025	Protein phosphatase 1c interacting protein 1 (*Ppi1*)	DUF4788, involved in protein phosphatase binding
DALD012340	FBgn0003460	sine oculis (*so*)	Homeobox gene regulating circadian rhythm, reproduction and development
DALD002312	FBgn0260861	TRAPP subunit 23 (*Trs23*)	Trafficking protein involved in vesicle-mediated transport
DALD007827	FBgn0033055	Tubulin-specific chaperone E (*Tbce*)	Regulates neurogenesis and neuromuscular synaptic transmission
DALD005587	FBgn0085362	Vitellin membrane-like (*Vml*)	vitellin membrane formation and dorsal/ventral axis specification
DALD009987	FBgn0015569	α-Esterase10 (*α-Est10*)	Regulator of imaginal disc-derived wing morphogenesis

### Heat stress depresses transcription more in the sensitive *D. hydei* than tolerant *D. buzzatii*

Mass bred progeny of wild-caught *D. hydei* and *D. buzzatii* females (an appropriate *D. aldrichi* strain not being available at that time) were subject to a set of pilot heat stresses to determine equivalent sublethal exposures. We found that 37.0 °C and 39.5 °C respectively were the maximum temperatures to which we could expose adults of the two species for 60 min without causing lethality. This difference between the two species’ maximum sublethal heat stress is consistent with previous reports on their relative thermotolerances (Kellermann et al. 2012b). We therefore carried out an experiment in the same generation in which we sampled cohorts of the two strains for transcriptomic analysis at the start, middle and end of their respective 60 min exposures to heat and then at five time points through the first 24 h of a recovery phase at 25.0 °C. The transcriptome analysis yielded 9109 genes from *D. hydei* for each of which we recovered a total of more than 50 RNA-Seq reads, and 9461 such genes from *D. buzzatii*. Of these, 1031 and 993 respectively were differentially expressed (DE) (FDR adjusted *p*-value < 0.05, log2 fold change in expression > 1 in at least two time points) relative to their pre-exposure level of expression.

Our first analyses of these data focused solely on the 10,443 1:1 orthologues in the two species that met the > 50 reads inclusion criterion above in either one of them. We found 123 of these showed DE in both species, 702 did so just in *D. hydei* and 681 did so just in *D. buzzatii*. This distribution represents about a 2 fold excess (123 cf. 63. 5; χ^2^ = 12.4, df = 1, *P* < 0.001) of shared DE responses compared to a null hypothesis of independent effects in the two species. However the majority of each species’ DE genes were not shared by the other species.

**Fig. 5:**
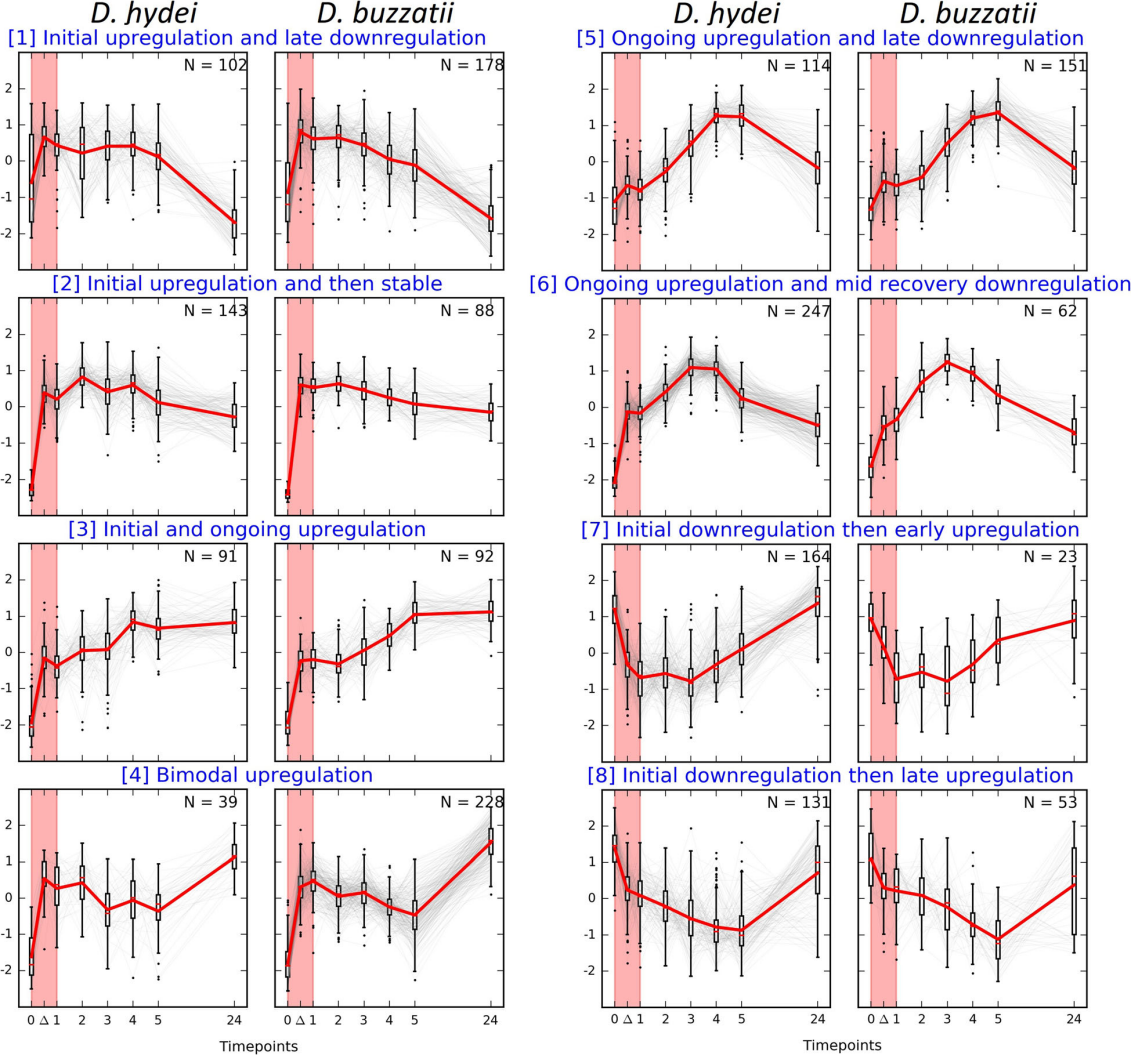
Fuzzy c-means clustering of genes that were differentially expressed (normalized values) in *D. hydei* and *D. buzzatii*. The y-axes are standardized expression values with mean = 0 and variance = 1

Our second analysis of the DE data used fuzzy c-means clustering of the temporal expression profiles of all 1031 DE genes in *D. hydei* and 875 of those in *D. buzzatii* (118 of the *D. buzzatii* DE genes showed erratic profiles which did not fit within any of the clusters formed) across all eight time points to organize them into eight clusters, representing eight major patterns of response over time (Fig. 5). Both species had genes in each expression trajectory cluster but the number of genes in most clusters varied considerably between the two species.

Two clusters (7 and 8) contained genes that were initially downregulated but subsequently recovered to around pre-exposure transcript abundances. *Drosophila hydei* genes greatly outnumbered *D. buzzatii* genes (combined across clusters, 219, or 288%, more genes) in both these clusters, even though *D. buzzatii* had been exposed to a 2.5 °C higher heat shock.

Genes in the other six clusters all showed initial upregulation, but each cluster showed a distinct trajectory through the recovery phase. Two clusters (3 and 5) containing the most similar numbers of genes from the two species are not considered further. Two others contained a large excess of *D. buzzatii* genes and were either downregulated relatively late in the recovery phase (cluster 1; 76, or 75%, more *D. buzzatii* genes) or showed a bimodal response with a second burst of upregulation late in the recovery phase (cluster 4; 189, or 485%, more *D. buzzatii* genes). Two clusters contained a large excess of *D. hydei* genes which showed little change through the recovery phase (cluster 2; 65, or 63%, more *D. hydei* genes) or ongoing upregulation followed by a mid-recovery phase downregulation (cluster 6; 185, or 298%, more *D. hydei* genes).

Overall there were over 700 genes from the discriminating clusters (ie excluding clusters 3 and 5) in excess in one or other of the species, indicating a profound difference in the transcriptional response of the two species to their respective ~ maximal sublethal high temperature shocks.

### Heat stress changes transcription of stress response genes in *D. hydei* and metabolic genes in *D. buzzatii*

We investigated the functions of the genes that differentiate the transcriptional responses of *D. buzzatii* and *D. hydei* in the heat shock experiment above by screening for enrichments of the GO sets and subsets above among the six discriminating expression trajectory clusters we had identified. Specifically we focussed our attention on the genes in the species in excess in each of these clusters which did not have orthologues in the other species in the same cluster (hereafter the discriminating genes).

The discriminating genes showed significant GO set enrichments (compared to the full gene set for the respective species) in four of the clusters, one with *D. buzzatii* genes in excess and three with *D. hydei* genes in excess (Fig. 3). The discriminating *D. buzzatii* genes in the initially upregulated Cluster 4 were enriched for sets F (Hydrolase activity), G (Primary metabolism) and H (Catalytic activity), suggesting upregulation of many metabolic enzyme activities in response to stress. Of the three clusters with *D. hydei* genes in excess, the discriminating genes in the initially upregulated cluster 2 were enriched for sets A (Cell component organisation and biogenesis), C (Development), S (Homeostasis) and W (Response to stimulus and stress); those in the initially upregulated cluster 6 were enriched for sets A and C again, plus B (Cell fate determination), I (Miscellaneous biological processes) and T (Receptor regulator activity); and those in the initially downregulated cluster 8 were enriched for sets K (Nucleotide sugars metabolism) and P (Metabolite and ion binding). The enrichments for these latter three clusters suggest impacts on a variety of fundamental cellular processes.

**Fig. 6:**
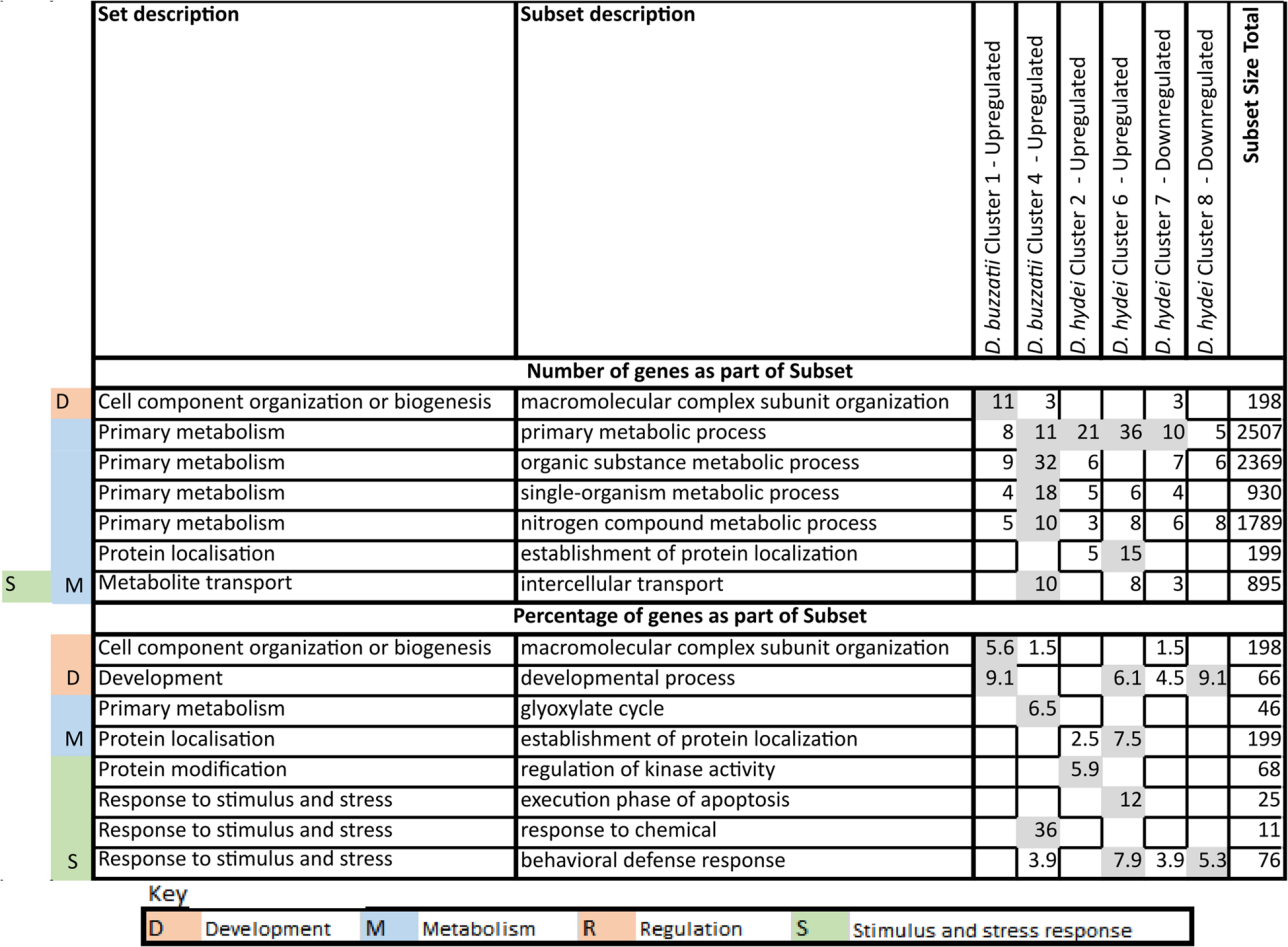
GO subsets most represented among the genes in the species in excess that lack orthologues in the same cluster in the other species in the fuzzy-c means clusters in the differential expression analyses. Representation is expressed in terms of either the total number (top) or percentage (below) of genes from the subset in question in the species in excess (again excluding any that also had orthologues in the other species in the same cluster). Only subsets containing more than 10 genes were analysed and only those where more than ten genes (top) or more than 5% (below) were involved in any one of the six clusters analysed are shown. These values are shaded. However for those subsets included on these criteria, any non-zero involvement in any of the other analyses are also shown, but not shaded. See the Methods section for further details of the methods used to generate this figure

Repeating these analyses at the subset-level (Fig. 6) showed enrichments in terms of absolute numbers and/or percentage increases for various metabolic, stress response and occasionally developmental terms. Of the two initially upregulated clusters with *D. buzzatii* genes in excess, the discriminating genes in cluster 1 were enriched, albeit weakly, for developmental and metabolic functions, and those in cluster 4 were quite strongly enriched for three metabolic functions and one stress response function. The discriminating genes in the initially upregulated clusters 2 and 6 which had *D. hydei* genes in excess were also enriched for metabolic and stimulus/stress response functions, but not the same ones as for cluster 4. The discriminating genes in the initially downregulated clusters 7 and 8 with *D. hydei* genes in excess were weakly enriched for various developmental, metabolic and stimulus/stress response functions.

Thus, overall, stress response functions were more often enriched in clusters where *D. hydei* genes were in excess, whether they were initially up- or downregulated. On the other hand, metabolic functions were more often enriched in the four initially upregulated clusters where *D. buzzatii* genes were in excess.

We also scrutinised the functions of individual genes contributing most strongly to the discrimination between the species in the six discriminating clusters. Specifically we looked at the ten genes showing the best fit (ie highest membership value) to the profile for each of those clusters in the species in excess which did not have orthologues in the other species in the same cluster (Table 4). The ten discriminating *D. buzzatii* genes contributing most strongly to each of the two initially upregulated *D. buzzatii*-dominated clusters principally implicated functions related to non-enzymatic responses to stimuli (cluster 1) and enzymes with potential roles in detoxification (cluster 4). The ten discriminating *D. hydei* genes contributing most strongly to each of the two initially upregulated *D. hydei*-dominated clusters principally implicated functions related to stress response (cluster 2) and regulation and stress response (cluster 6). The ten discriminating *D. hydei* genes contributing most strongly to each of the two initially downregulated *D. hydei*-dominated clusters principally implicated functions related to regulation (cluster 7) and development (cluster 8), with both clusters also implicating stress response genes. Thus all four *D. hydei*-dominated clusters involved various stress response genes but such genes were not prominent in either of the *D. buzzatii*-dominated clusters. This agrees well with the findings from the set and subset enrichment analyses above.

**Table 4:** Top 10 genes driving differential expression in six most divergent clusters between *D. hydei* and *D. buzzatii*, along with the membership coefficient ranges of the genes in parentheses*.* Functional information for each gene was summarised based on Flybase descriptions for *D. melanogaster* orthologues, where available, and otherwise, domain classification of the protein sequence using gene ontology and Pfam family annotations

Gene ID	Gene name	Gene function
Cluster 1: Initial upregulation & late downregulation (0.51, 0.59); *D. buzzatii* in excess, mainly response to stimuli		
DBUZO2014318	Odorant receptor 10a (*Or10a*)	Chemoreceptor that mediates response to volatile chemicals
DBUZO2013263	Ucp4C (*Ucp4C*)	Protein uncouples respiration and energy dissipation
DBUZO2011185	CG17387	Involved in cilium dependent cell motility
DBUZO2010006	CG11475 (*DUF89*)	May be involved in protein methylation in response to DNA damage
DBUZO2009533	*Ppm1*	Involved in protein serine/threonine phosphatase activity
DBUZO2005585	CG9702	Transmembrane transporter involved in sulfate transport and regulation of intracellular pH
DBUZO2002166	NA	
DBUZO2001878	Gustatory receptor 97a (*Gr97a*)	Involved in sensory perception of taste
DBUZO2001291	CG5538	Voltage gates chloride channel
DBUZO2001235	NA	
Cluster 2: Initial upregulation & then stable (0.41, 0.45); *D. hydei* in excess, mainly stress response		
DHYD012352	Heat shock protein 83 (*Hsp83*)	Heat shock protein 90 family, regulates response to abiotic stress and circadian behaviour
DHYD011340	NA	Heat Shock protein 70 family
DHYD011253	CG14516	Zing ion binding peptidase
DHYD011218	frayed (*fray*)	Protein serine/threonine kinase involved in several development processes
DHYD007159	Egg-derived tyrosine phosphatase (*EDTP*)	Protein-tyrosine phosphatase-like gene involved in oogenesis and dephosphorylation
DHYD006916	CG8944	DNA binding zinc finger domain gene
DHYD005532	NA	
DHYD003416	CG4950	
DHYD001898	tramtrack (*ttk*)	DNA binding RNA polymerase promoter involved in response to external stimulus
DHYD000023	Cyclin E (*CycE*)	Cyclin dependent protein kinase, positively regulates cell cycle and morphogenesis
Cluster 4: Bimodal upregulation (0.69, 0.76); *D. buzzatii* in excess, mainly enzymes involved in detox		
DBUZO2012733	NA	Cytochrome P450
DBUZO2012084	NA	S1 peptidase
DBUZO2011594	CG3699	Short-chain dehydrogenase/reductase, involved in fatty-acid beta-oxidation
DBUZO2009194	NA	Cytochrome P450
DBUZO2005738	CG31087 (*DUF227*)	Involved in neurogenesis
DBUZO2005447	alpha-Esterase-5 (*alpha-Est5*)	Type B carboxylesterase
DBUZO2002940	Maltase A4 (*Mal-A4*)	Glycosyl hydrolase, involved in carbohydrate metabolism
DBUZO2002034	Tetraspanin 42 Eq (*Tsp42Eq*)	Cell surface receptor
DBUZO2001132	CG42335	M1 peptidase involved in proteolysis
DBUZO2000745	CG31198	M1 peptidase involved in proteolysis
Cluster 6: Ongoing upregulation &-mid-recovery downregulation (0.50, 0.53); *D. hydei* in excess, mainly regulation & stress response		
DHYD014524	Hira (*Hira*)	Histone H3-H4 chaperone, involved in various replication-independent nucleosome assembly processes
DHYD014213	ellipsoid body open (*ebo*)	Complexes with actin, chic, and Ran-GTPase to mediate actin nuclear export
DHYD014093	Ajuba LIM protein (*jub*)	Protein inhibits activation of the Hippo pathway kinase
DHYD011516	CG7065	
DHYD011485	maroon-like (*mal*)	Molybdenum cofactor sulfurase involved in ommochrome biosynthesis
DHYD010172	slow as molasses (*slam*)	Involved in protein localisation and migration
DHYD008182	withered (*whd*)	Involved in response to starvation, ethanol, oxidative stress and metal ions
DHYD007200	Enigma (*Egm*)	Involved in response to endoplasmic reticulum stress; fatty acid beta-oxidation; lipid homeostasis and cellular response to oxidative stress
DHYD006980	CG8611	Involved in unwinding RNA secondary structure
DHYD001526	Hillarin (*Hil*)	W180-domain protein, affects cytokinesis, developing nervous system
Cluster 7: Initial downregulation & early upregulation (0.42, 0.44); *D. hydei* in excess, mainly regulation & stress response		
DHYD015984	Catalytic subunit 3B of the oligosaccharyltransferase complex (*Stt3B*)	Involved in post-translational protein modification
DHYD015297	*Ror*	Involved in protein phosphorylation; central nervous system development and transmembrane receptor protein tyrosine kinase signalling pathway
DHYD012423	Myocardin-related transcription factor (*Mrtf*)	Transcription factor with roles in cell migration during development
DHYD009550	pasilla (*ps*)	A nuclear RNA binding protein implicated in splicing
DHYD008649	epithelial membrane protein (*emp*)	Associated with autophagic cell death
DHYD006908	NA	
DHYD006727	NA	Involved in fatty acid elongation
DHYD004130	CG31522	Involved in fatty acid elongation
DHYD004039	Heterogeneous nuclear ribonucleoprotein at 87F (*Hrb87F*)	Involved in regulation of alternative mRNA splicing and responses to starvation and heat
DHYD001282	CG16713	Pancreatic trypsin inhibitor involved in development and signalling
Cluster 8: Initial upregulation & late upregulation (0.40, 0.43); *D. hydei* in excess, mainly development & stress response		
DHYD018935	short stop (*shot*)	Cytoskeletal linker molecule in the nervous system and other tissues
DHYD016713	Gp150 (*Gp150*)	Transmembrane glycoprotein; regulates Notch signalling in development
DHYD016696	acyl-Coenzyme A oxidase at 57D distal (*Acox57D-d*)	fatty acid beta-oxidation
DHYD014956	rugose (*rg*)	Involved in olfactory learning and short-term memory
DHYD012466	Laminin A (*LanA*)	Regulates growth and locomotion of cells
DHYD009427	Glutathione S transferase Z2 (*GstZ2*)	Involved in aromatic amino acid and glutathione metabolic process
DHYD009383	CG43222	
DHYD001387	*Not1*	Muscular development, morphogenesis, mRNA catabolic processes
DHYD001006	CG42500	Induced during the immune response of Drosophila
DHYD000649	NA	

None of the 60 genes contributing most strongly to the discrimination between species in the six discriminating clusters above were among the orthogroups generated in the cactus use branch or those generating inparalogues or under positive selection in the *D. aldrichi* branch.

## Discussion

We have found an unusually high frequency of gene gain events in internal branches of the *repleta* species group phylogeny during which the topology of our phylogenies suggests the ability to use cactus hosts was acquired. The frequency of gene gains also remained quite high in the subsequent cactus specialisation branch, which was associated with the lost use of other hosts but the gain of relatively high heat and desiccation tolerance. It seems more likely that the gene gains in this branch were associated with the gain of climatic tolerance than the narrowing of host range; a loss of function seems more likely to be linked to gene loss rather than gene gain [50]. The rate of gene gains then slowed considerably in the following *mulleri* complex branch, before picking up again in certain terminal branches, in particular the *D. aldrichi* branch, as the species diverged in their use of different cactus hosts and in climatic tolerance and *D. aldrichi* evolved the highest level of heat and desiccation tolerance.

There is no direct precedent for our analytical approach to assessing rates of accepted duplication events during adaptive evolution. There are many cases where high numbers of duplication events for specific genes or gene families have been associated, in several cases causally, with host shifts [34, 51, 52], or the development of insecticide resistance, in insects [53, 54]. However our data show for the first time a suite of duplications across a range of genes, families and physiologies associated with adaptive changes. Further they do so for both internal and terminal branches of the phylogeny.

We did not find unusually high frequencies of positive selection events in the early branches of the phylogeny with the high frequency of gene gains, but did so in the terminal *D. aldrichi* branch and overall we found a positive association between the specific genes involved in duplication events and those subject to positive selection in the later branches. Our findings are broadly consistent with the hypothesis that the evolution of new functions is associated with gene duplication and subsequent neofunctionalisation events ([55], but also see [56]).

A broad range of biochemical functions were implicated both in the evolutionary changes above and in the changes in other branches associated with the cactophilic species which did not show such unusually high rates of change. Notably Gene Ontology terms associated with the metabolism of organic and nitrogenous compounds were enriched in a number of branches associated with cactophilism. The nitrogen and secondary compound constitutions of rotting cacti differ markedly from those of rotting fruits and these enrichments are consistent with the idea that some of the evolutionary changes were causally linked to changes in host use. They are also consistent with previous comparative genomic and transcriptomic studies of host races of the cactophilic *D. mojavensis* and *D. mettleri* which also associated differences in host use with such biochemical functions [12, 14–16].

We found that gene gains in the relatively long ancestral *repleta* group branch were enriched for Gene Ontology terms associated with a particularly wide range of metabolic functions. This may relate to the wide array of host uses that evolved in various daughter lineages, ranging from the cactophiles in the *hydei* subgroup of interest here to the flower feeders in the *bromeliae* subgroup, the independently evolved cactophilism in the *nannoptera* subgroup, the fungus feeders in the *fasciola* subgroup and animal faeces in *D. repleta* itself.

A range of functions were also enriched among the disproportionately high number of genes generating inparalogues and/or under positive selection in the terminal *D. aldrichi* branch, but the twenty genes contributing most strongly to the positive selection events in this branch predominantly involved regulatory and developmental processes. These events may contribute to the extreme heat and dessication tolerance of this species.

We also carried out a comparative transcriptome analysis on freshly caught collections of another heat tolerant species, *D. buzzatii*, and the relatively heat sensitive *D. hydei* in order to further investigate the potential molecular basis of the thermal tolerance differences in the *repleta* group. Several hundred genes were found to respond differently to comparable levels of sub-lethal heat stress in the two species. Even though the heat shock administered to *D. hydei* was less extreme in terms of temperature than that given to *D. buzzatii*, *D. hydei* genes more often showed initial downregulation responses than did *D. buzzatii* genes and more of their responsive genes had orthologues associated with stress responses in other species. However, there was no significant overlap between the heat responsive genes in either species and those implicated in the gene gain and positive selection events in *D. aldrichi*, suggesting that the genes showing different transcriptional responses to heat shock in the two *repleta* group species tested are not those on which natural selection has acted to confer extreme heat tolerance in *D. aldrichi*. Interestingly, there was also no significant overlap (data not shown) between the heat responsive genes in either species and a panel of candidate genes associated with heat stress responses in intraspecific comparisons in *D. melanogaster* [17, 18]. This could be because of differences in methodologies, the very different ecologies of the different species, or the phylogenetic distance between *D. melanogaster* and the *repleta* group species studied here.

Finally we note that hundreds of genes were implicated in the disproportionately high rates of gene gains and positive selection events associated with the phenotypic changes above. This suggests large-scale genomic changes underlay the phenotypic evolution, which is consistent with the findings of the previous comparative analyses involving the cactophilic *repleta* group species ([5, 14–16] and see Background). The scale of change specifically associated with the cactus use branch is also consistent with the scale of nucleotide differences found in reduced representation genomic analyses of host shifts in *Rhagolites* flies [57], but it is greater than those so far associated with such analyses of host shifts in *Timema* stick insects [58] or in full genome analyses of adaptation to the otherwise toxic host *Morinda citrifolia* in *D. sechellia* and some *D. yakuba* [59, 60]. In so much as the rate of adaptation to the anthropogenic climate change now in prospect may be limited by the rate at which new duplication and mutation events are generated, the scale of change in the internal cactus specialisation and terminal *D. aldrichi* branches associated with increased climatic tolerance also suggests it would be beyond the capacity of many current species to achieve equivalent shifts in climate niches in the timeframes now projected for climate change.

## Conclusion

Our phylogenomics analysis of fourteen *Drosophila* species finds bursts of duplication and selection events associated with adaptation to arid environments. These adaptations include both acquisition of cactophilic host use and greater tolerance of climatic extremes. The bursts of duplication and selection, which support the duplication and neofunctionalisation theory of adaptation, have occurred across both internal and terminal branches of the phylogeny encompassing 6–8 million years. The genes involved cover a wide range of physiological functions and there is little overlap between them and the genes whose transcriptional profiles after heat stress differ between the two species.

## Methods

### Fly strains

The *D. hydei* and *D. repleta* strains used for genome sequencing were collected from Townsville, QLD, Australia and Wandin, VIC, Australia, respectively and then inbred in the laboratory for 17 generations of single pair full-sibling mating to reduce their heterozygosity (expected inbreeding co-efficient > 0.7 [61]). The *D. aldrichi* strain sequenced was originally caught in Mexico in 2002 and was likely inbred to some degree while being maintained in the University of California San Diego Drosophila Species Stock Center (stock number: 15081–1251.13). It was further inbred for two generations of sibling mating from a single pair of flies prior to sequencing.

Individuals for the mixed life stage transcriptome sequencing that we used to augment the annotations were obtained from the inbred *D. hydei* and *D. repleta* lines above and from a wild-caught mass bred line of *D. aldrichi* from Inglewood, QLD, Australia. RNA was prepared from a mixture of six life stages (embryos, first, second and third instar larvae, pupae and adults, combined in approximately equal weight amounts) for each species.

Recently collected strains of *D. hydei* and *D. buzzatii* were used in the thermal stress transcriptomics experiment*.* The offspring of 50 field females were pooled to establish a mass bred population for each species*.* The *D. hydei* females were collected from Pascoe Vale, Melbourne, VIC and the *D. buzzatii* from Inglewood, QLD, Australia. The mass bred strains were maintained at 25 °C for one generation prior to the experiment.

### Genome sequencing and assembly

#### Sequencing

Adult females from the inbred *D. repleta*, *D. hydei* and *D. aldrichi* strains were harvested and DNA extracted from heads to minimise contamination with DNA from their gut flora. Six libraries were then prepared from the DNA from each species for sequencing on an Illumina HiSeq2000 platform; three paired-end libraries spanning 250 bp, 500 bp and 800 bp using 150 bp or 100 bp paired-end sequencing runs, plus three long insert mate-pair libraries with insert sizes of 2kbp, 5kbp and 10kbp using 49 bp paired-end runs. All library preparation and sequencing were carried out at Beijing Genomics Institute (BGI), Shenzhen. At least 35Gb of raw sequence data were obtained per species, yielding a final read coverage of ~210x of the estimated genome size.

#### Assembly

The raw paired-end and mate-pair reads were assessed using FastQC [62] and novel contaminant sequences identified using K-mer counting with Jellyfish [63] to provide inputs for trimming and filtering using Trimmomatic [64].The insert size for each library was then re-estimated by aligning the trimmed reads to the *D. mojavensis* genome [see Additional file 1: Text S2]. Quorum [65] was then used to correct the trimmed reads for *D. hydei* and *D. repleta* while BFC [66] was used for *D. aldrichi* (because higher levels of heterozygosity were expected for this species)*.* The corrected reads were then assembled using the MaSuRCA assembler [67] and contigs extended and scaffolded using the SSPACE 2 scaffolder [68].

The scaffolded genomes were further improved by local realignment and gap-filling carried out using multiple rounds of Pilon [69], with reads aligned using SNAP aligner [70], until no iterative gains were observed. The final assemblies were then benchmarked using the BUSCO pipeline [29] following the authors’ instructions.

#### Repeat sequence analysis

Tandem repeats and transposable elements (TEs) in *D. hydei*, *D. repleta, D. buzzatii* and *D. aldrichi* were identified following the strategy of Zhang et al. [71]. TRF [72] and RepeatMasker [73] were used to characterise tandem repeats; RepeatMasker was used with the Repbase [74] database of known repeat sequences in *D. melanogaster* to identify repeats shared with the latter species; and previously undescribed repeats were identified using LTR_FINDER [75], PILER [76] and RepeatScout [77]. Repeat proteins were also identified using RepeatProteinMask (version 3.2.2) as implemented in RepeatMasker. All the repeat sequences in each species identified by the different methods were combined into a final repeat library and categorized in a hierarchical way, as per [71].

### Genome annotation

#### Transcriptome sequencing for gene prediction

We made RNA-Seq transcriptomes from mixed life stage RNA preparations of *D. repleta*, *D. aldrichi* and *D. hydei* on the Illumina HiSeq 2000 platform to assist in gene annotations. The RNAs were prepared using the Zymo Direct-zol™ RNA extraction kits (Zymo Research, Irvine, USA). A standard RNA-Seq library was prepared for *D. hydei*, while strand-specific libraries were prepared for *D. repleta* and *D. aldrichi* according to Borodina et al. [78]. All three libraries were prepared by BGI following standard Illumina protocols. Hundred bp unstranded paired-end RNA-Seq sequencing yielded 4Gb of data for *D. hydei* and 3Gb each for *D. repleta* and *D. aldrichi.* These data were then filtered to remove reads which had more than 10% of bases unknown, more than 40 bases with Phred scores less than 7, or > 20% of the sequence comprising adaptor sequence. This left a high quality read set of ~ 2 Gb for *D. hydei* and *D. repleta* and ~ 3 Gb for *D. aldrichi*.

#### Annotation of gene models

We identified, evaluated and collated protein coding and putative non-coding genes in *D. aldrichi, D. buzzatii, D. hydei* and *D. repleta* using ten sets of RNA-Seq data plus homologue-guided and ab-initio based gene prediction as detailed in Additional file 1: Text S2. The repeat sequences were first used to soft-mask the genome, upon which we mapped 2315 single copy *D. melanogaster* genes (248 using CEGMA [28] and 2067 from OrthoDB7 [79] using Exonerate [80]) and the *D. melanogaster* and *D. mojavensis* proteomes [80] to generate splice-aware alignments.

The resulting splice junctions were used to guide two-pass mapping of RNA-Seq data onto the respective genomes using GMAP/GSNAP (v 2014-08-20; [81] and STAR aligner (v 2.4.0; [82]. The RNA alignments were then used to annotate transcripts using StringTie [83] and genome-guided Trinity [84], while trimmed RNAseq data were used for de novo assembly of the species’ transcripts using Trinity. All three transcript sets were collated using PASA2 [85] to create a comprehensive transcript database and high-quality training dataset (GTrainSet) for training and subsequent annotation of genes using four ab-initio predictors; Genemark-ET [86], Glimmer [87], Augustus (v 3.0.2; [88] and SNAP [89].

All ten sets of gene models created were finally passed to EvidenceModeler [90] to create a comprehensive whole genome annotation. Greatest weight was given to StringTie (15) followed by GTrainSet (12) and homology-based (11) evidence, and least to the ab initio predictions (2–4). The gene set was then updated to annotate alternative splice events, 3′ and 5’ UTRs and to refine gene boundaries using RNA-evidence in PASA2. Finally, nested genes were added using the homology matches in *D. melanogaster* and *D. mojavensis*, creating the official gene sets (OGSs).

### Comparative genomics

#### Classification of orthologues

The Orthonome pipeline (http://www.orthonome.com/; [24, 91]) was applied to sets of 1:1 orthologues and orthogroups extracted from the OGSs. The pipeline also identified gene birth events and inparalogues. Only the longest isoform for each gene in each genome was used in this analysis. Note that while Orthonome partitions the output into orthologues, inparalogues and gene births, the number of inparalogues will be inflated to a degree by artefactual inparalogues – which are biologically orthologous genes where one member is missing from the data or where the duplication event occurred so close to a speciation event that it could not be distinguished from two independent post-speciation duplication events.

Three Orthonome analyses were carried out, the first for calculating the phylogeny and the remaining two for comparative genomics. These were (i) one using the twelve Flybase genomes [92] (*D. ananassae, D. erecta, D. grimshawi, D. melanogaster, D. mojavensis, D. persimilis, D. pseudoobscura, D. sechellia, D. simulans, D. virilis, D. willistoni and D. yakuba*), eight modENCODE genomes (https://www.hgsc.bcm.edu/arthropods/drosophila-modencode-project; *D. biarmipes, D. bipectinata, D. elegans, D. eugracilis, D. ficusphila, D. kikkawai, D. rhopaloa and D. takahashii*) and our newly annotated *D. aldrichi, D. buzzatii, D. hydei* and *D. repleta* genomes, (ii) one using this set minus the eight lesser quality modENCODE genomes, and (iii) one just using the five *repleta* species and *D. melanogaster*.

#### Species phylogeny

The orthologues obtained from analysis (i) above led to the identification of 4935 orthogroups. The orthogroups were then filtered to keep only those that satisfied the following criteria: (1) none of the species had any traceable duplications (i.e. best blast hit was always an orthologue), (2) relatively slowly evolving, with no genes presenting a Ka/Ks > 1 (see below for calculation method), (3) > 200 amino acids in length (unless all members of the orthogroup were within five residues length of each other), and (4) all genes within a 1.5 median absolute deviation from the median length of the orthogroup. The nucleotide coding sequences from the 1802 orthogroups retained were used to construct both a concatenated species phylogeny using FastTree2 [39] with 1000 internal boot-strap replicates and a consensus tree using IQ-Tree [32] allowing a free rate model and with each gene treated as its own partition. The two phylogenies had the same topology but the IQ-Tree tree was chosen for all subsequent analyses. Divergence time was estimated with MEGA7 (Rel-Time) [93] based on a calibration for Drosophila obtained according to Obbard et al. [94].

We also constructed two other trees to interrogate more closely the *D. hydei – D. repleta* relationship. One was another IQ-Tree based tree and the other was a *BEAST2 Bayesian tree [40]. Both were constructed using the inferred amino acid sequences for the 100 best genes (< 1% gappyness and no compositional heterogeneity; χ^2^*p*-value > 0.05). Concordance factors [41] and partitioned coalescence support scores [42] were then used to compare the support for the different phylogenies, finding the *BEAST2 tree performed less well than the others on both measures.

#### Functional annotations

We first augmented the functional annotations for *D. melanogaster* genes obtained from FlyBase using InterProScan 5 (January 2016 release) [30]. These updated annotations were transferred to *D. aldrichi, D. buzzatii, D. hydei and D. repleta* based on orthologous relationships estimated using Orthonome (see above). Functional annotation of the inparalogues remaining was carried out by identifying the phylogenetically closest orthologue or inparalogue from *D. melanogaster* while de novo annotation was carried out for gene births using InterProScan 5. Gene interaction networks were also transferred from *D. melanogaster* using orthology [95]. FlyBase Gene Group annotations were transferred to the *repleta* species in a similar fashion.

#### Evolutionary rate analyses

Branch-site model tests (adaptive branch-site random effects likelihood; aBSREL) were carried out to identify specific lineages in the phylogeny (both terminal and internal branches) in which orthogroups identified by Orthonome may have been under positive selection. This analysis was carried out using all the FlyBase genomes and the four *repleta* group genomes annotated in the current study. We assumed fixed branch lengths based on the phylogeny constructed above and calculations used the HYPHY package [48]. Each branch in the *repleta* lineage was analysed and a False Discovery Rate- (FDR-) corrected χ^2^*P*-value cut-off of < 0.05 was used.

As per O’Toole et al. [49], the terminal branches were also analysed to compare the proportions of genes under positive selection in the branches that had versus had not generated inparalogues.

### Comparative thermal stress transcriptomics for *D. hydei* and *D. buzzatii*

The goal of this experiment was to compare the transcriptional responses to thermal stress of species that differed in high temperature tolerance. Mass-bred populations initiated from recently-collected flies were used for these experiments to minimise issues with changes in gene expression during laboratory adaptation. We also conducted pilot studies on these mass bred populations to identify the most suitable testing temperatures (see Additional file 1: Text S2 for details). These were found to be 37 °C for *D. hydei* and 39.5 °C for *D. buzzatii*, consistent with previous findings of their differences in thermal tolerance [3].

For the stress assays, three independent replicates, each of ten virgin adult females aged two days since emergence, were harvested at each time point indicated in Additional file 2: Figure S3 and snap-frozen in liquid nitrogen. One time point was frozen immediately prior to exposure (used as the control for differential expression comparisons), one half way through the exposure, one immediately after the exposure and five at defined intervals over the next 24 h. The snap-frozen material was stored at − 80 °C until RNA extraction (pooling all ten flies in each replicate) using the Zymo Direct-zol™ RNA extraction kits (Zymo Research, Irvine, USA). RNA-Seq libraries were prepared and sequenced with the Illumina HiSeq 2000 platform as above. All 48 libraries (8 time points and 3 replicates for each species) were run on the same instrument in the same flow cell.

The sequence reads for each species were filtered for low quality and adapter sequences and trimmed with Trimmomatic [64] as above. The cleaned reads were then aligned to their respective genomes using a two-pass strategy in STAR aligner (v 2.4.2a) [82], which also produced the read counts matrix for each alignment. These outputs were combined to create an expression value matrix for each gene across all eight time points as input for subsequent analyses.

Counts for all genes with more than 50 reads (threshold for genes with low expression) were normalised across samples using the trimmed mean of M-values method [96] and converted to log2 counts per million values (log2cpm) with associated quality weights using the voom-limma pipeline [97]. Batch effects due to manual handling were corrected with ComBat [98] against an intercept only model. Changes in gene expression over time were then modelled in limma. Genes with a significant difference in expression relative to the first time point, ie the one immediately prior to the treatment, (FDR) adjusted *P* value less than 0.05 and a log2 fold-change in expression greater than 1) in at least two of the subsequent time points were considered to be heat responsive.

To identify common patterns of expression among differentially expressed genes, the expression of each gene across the different time points was first standardised (independently in each species) to have a mean of 0 and standard deviation of 1. The standardised expression values for both species were then combined and subjected to fuzzy c-means clustering [99]. Trials with from four to 20 clusters yielded the highest fuzzy means partitioning coefficient with eight clusters, so further analysis was based on those eight clusters. Membership exponent values were then calculated for each gene in each cluster to identify the genes best explaining the cluster pattern across time points. The expression patterns for the genes with the highest membership exponent in each cluster were then plotted to visualise trends. The core genes of each cluster (membership value greater than 0.5) were used for the functional enrichment analysis below.

### Functional enrichment analyses

Functional enrichment of gene ontologies was carried out after first clustering the 47,676 GO terms ([31]; release: 2016-11-03) into 43 mutually exclusive sets using a Louvain clustering method [100] and allocating semantic similarities as edge weights between connected hierarchical GO terms. We used 23 of these sets that represent biological processes as the framework for our enrichment analyses. Additional file 2: Table S5 summarises the constitution of these sets in terms of the major subsets within them. For each analysis, the respective gene lists were tested for enrichment of each GO term based on frequency with Goatools (https://github.com/tanghaibao/goatools) and the GO terms obtained as significantly enriched were then compressed using REVIGO [101] and assigned to the sets and subsets above. The 23 sets were given names which summarised the dominant highest level GO term(s) within them, while the subsets simply took the names of the dominant highest level GO term (which was always a lower level than in the sets) within them.

## Additional files

Additional file 1: **Text S1.** Phylogenetics of sophophoran subgenus. **Text S2.** Detailed materials and methods for genome assembly and annotation. (DOCX 49 kb)

Additional file 2: **Figure S1.** Overlap between the orthogroups generating inparalogues in the different species. **Figure S2.** Overlap between orthogroups belonging to genes under positive selection in each *repleta* group species. **Figure S3.** Scheme for heat stress and recovery assay. **Table S1.** Scaffold and contig length based statistics as well as results from BUSCO genome assessment using the dataset for the five assembled *repleta* group genomes and *D. melanogaster.* **Table S2.** Repeat content analysis for the six species analysed in this study, characterising transposable elements as well as tandem repeats. **Table S3.** Genome annotation statistics for the five assembled *repleta* group genomes and *D. melanogaster*. **Table S4.** Number of pairwise orthologues between *D. melanogaster* and the five *repleta* species, plus the previously published annotation of *D. buzzatii*. **Table S5.** Sets of functional terms describing hierarchical grouping of gene ontology terms and the number of genes in *Drosophila melanogaster* within each set. (DOCX 301 kb)
