# Supplementary material for: Genomic changes associated with adaptation to arid environments in cactophilic Drosophila species
Source: BMC Genomics. 2019 Jan 16;20:52. doi: 10.1186/s12864-018-5413-3 (PMC6335815; doi:10.1186/s12864-018-5413-3)
Supplement: Supplementary file 1 — Text S1. Phylogenetics of sophophoran subgenus. Text S2. Detailed materials and methods for genome assembly and annotation. (DOCX 49 kb) [file 12864_2018_5413_MOESM1_ESM.docx]

**Text S1**

**Phylogenetics of the sophophora subgenus**

We constructed a genome-based phylogenetic tree using 1,802 single copy 1:1 orthologues (see Materials and Methods) across 24 species (*D. aldrichi, D. ananassae, D. biarmipes, D. bipectinata, D. buzzatii, D. elegans, D. erecta, D. eugracilis, D. ficusphila, D. grimshawi, D. hydei, D. kikkawai, D. melanogaster, D. mojavensis, D. persimilis, D. pseudoobscura, D. repleta, D. rhopaloa, D. sechellia, D. simulans, D. takahashii, D. virilis, D. willistoni and D. yakuba*).

In addition to resolution of the basal node for the repleta group, the phylogeny also resolved more phylogenetic relationships than earlier studies [1]. Specifically, the phylogeny shows *D. elegans* and *D. rhopaloa* as a pair of sister species and *D. ficusphila* as the sister clade to all *Sophophorans* in our sample except the *montium* and *ananassae* subgroups (as opposed to just *D. elegans* and *D. rhopaloa*). In addition, our phylogeny shows the clade containing the *takahashii* and *suzukii* subgroups to be the sister clade to that containing the *eugracilis* and *melanogaster* subgroups with more confidence than previously possible.

**Text S2**

**Materials and methods**

***Genome assembly***

The quality of the raw sequence data for each species was initially assessed using FastQC [2], plus K-mer counting with Jellyfish [3] to identify novel contaminant sequences. Trimmomatic [4] was then used to remove low quality reads and trim other reads to remove bases with Phred scores < 9 and contaminant and/or adaptor sequences attached to reads. Only paired reads where both sequences retaining >80% of the presumptive original size were retained for the genome assembly and the rest were marked as singletons for genome correction (see below). Approximately 5% and 10% of the paired-end (short insert) and mate-pair (long insert) raw data respectively were put aside during these quality control steps. The trimmed reads were then aligned to the published *D. mojavensis* genome (which BUSCO and assembly N50 stats indicated was better assembled than the published *D. buzzatii* genome; see Table 1 above) to re-estimate the insert size distributions for each library. We generally observed a 10% reduction in insert sizes compared to the original size selection; this reduction was then incorporated into the assembler parameters below.

The trimmed reads for *D. hydei* and *D. repleta* were corrected for sequence polymorphism using Quorum [5], but BFC [6] was used to assist in stringent heterozygosity-sensitive read correction for *D. aldrichi* (because higher levels of heterozygosity were expected for this species)*.* Corrected reads were assembled with the MaSuRCA assembler [7], which is based on but extends the Celera CABOG assembler [8]. K-mer values of 105bp and 37bp for paired-end and mate-pair data respectively were used as input parameters into MaSuRCA. MaSuRCA first assembles super-reads by extending reads using best match or sequence overlap and then assembles contigs using CABOG. We modified this protocol to utilise only paired-end data for super-read assembly, but then used all the data for contig assembly. All contigs > 230bp (the observed insert size for our smallest short-insert library) were then joined into scaffolds using the super-reads and Illumina sequencing data as input for the SSPACE 2 scaffolder [9]. We also allowed SSPACE to extend ends of scaffolds using unused raw sequence data where possible. Once again we restricted the minimum scaffold size retained to 230bp.

***Local reassembly and gap filling***

The scaffolded *D. hydei*, *D. repleta* and *D. aldrichi* genomes were further improved by local realignment and gap-filling with multiple iterations of Pilon [10]. For this purpose, we used corrected reads, MaSuRCA super-reads and singletons that had been removed during raw sequence processing as input data. SNAP aligner [11] was used for accelerated alignment of these sequences to the scaffolded genome with only the best alignment of every sequence utilised as input for correction. For every genome, multiple iterations of Pilon were carried out until improvements affected less than 5% of base pairs in the previous iteration. Thereafter, all remaining reads were *de novo* assembled into new scaffolds. The final genome assemblies were then benchmarked for quality assessment using the BUSCO pipeline [12] following the authors’ instructions.

***Annotation of gene models***

We created a novel genome annotation pipeline to identify, evaluate and collate protein coding and putative non-coding genes through use of the RNA-Seq data and homologue-guided and *ab initio* gene predictions. For each species, we first utilised the repeat annotations above to soft-mask the genome. For the homologue-guided predictions, we then assembled a dataset of 2,315 *D. melanogaster* genes (248 from CEGMA [13] and a further 2067 from OrthoDB7 [14]) that have only 1:1 orthologues across at least 90% of the arthropod genomes so far catalogued. Splice-aware annotations of their orthologues in the newly assembled genomes were then constructed using the CEGMA [13] and Exonerate [15] pipelines and the published *D. melanogaster* and *D. mojavensis* proteomes [15].

The splice junctions identified above using the 2,315 *D. melanogaster* genes were used to guide the first pass of a splice-aware two-pass alignment of the cleaned RNA-Seq data from *D. hydei*, *D. repleta* and *D. aldrichi* to their respective genomes with GMAP/GSNAP (v 2014-08-20; [16]) and STAR aligner (v 2.4.0; [17]). The second pass repeated the process using updated splice junction databases from the first pass. The resultant alignments were then used to identify contiguous transcript sequences with StringTie [18] and genome-guided Trinity [19]. We also identified transcripts using *de novo* assembly of RNA-Seq data with Trinity. The *de novo* and genome-guided transcriptomes were then collated using PASA2 [20] to create a comprehensive transcript database for each species and to identify complete RNA-Seq guided gene models with Transdecoder (https://transdecoder.github.io/).

The Transdecoder gene models that had >80% sequence length and identity match to *D. melanogaster* genes (including the 2,315 genes above) were combined to prepare a golden training set (GTrainSet) for *ab initio* prediction of genes. The GTrainSet splice junctions were then augmented with the self-training Genemark-ET gene predictor [21], after which the updated GTrainSet and gene, UTR and exon length parameters were used to train and run *ab initio* predictors in Glimmer [22], Augustus (v 3.0.2; [23] and SNAP [24]. The gene, UTR and exon length parameters were recalibrated for the GTrainSet genes after every *ab initio* prediction run. All *de novo* predictions from the four predictors were then screened to remove small genes (translated peptide < 35 amino acids).

All ten sets of gene models created above - two RNA-Seq based (PASA2/Transdecoder and StringTie), five *ab initio* (CEGMA, Genemark-ET, Glimmer, SNAP and Augustus), two homology-guided (*D. melanogaster* and *D. mojavensis*) and the GTrainSet - were finally passed to EvidenceModeler [25] to create a comprehensive whole genome annotation. Greatest weight was given to RNA-Seq (weight of 15) followed by GTrainSet (12) and homology-based (11) evidence and least to the *ab initio* predictions (2-4). The gene set collated by EvidenceModeler was then updated to annotate alternative splice events and 3’ and 5’ UTRs, and to refine gene boundaries using RNA-evidence in PASA2. This set was processed to add nested genes using the homology matches in *D. melanogaster* and *D. mojavensis*, creating the official gene set (OGS).

The above pipeline was also used to re-annotate the published *D. buzzatii* genome [26], which we found was lacking many otherwise conserved gene models in the first run of our orthology analysis (see below). In the absence of a mixed life stage transcriptome for this species, we used our own stress transcriptome data (detailed below) for this re-annotation.

***Pilot studies to identify suitable temperatures for thermal stress transcriptome***

We also conducted pilot studies on these mass bred populations to identify the most suitable testing temperatures. This was done by exposing ten replicates of ten adult females each to a range of high temperatures for 60 minutes and then returned them to 25°C, recording their survival through the stress and for the following 24 hrs, so that sub-lethal temperatures could be identified. *Drosophila hydei* was tested from 35°C to 39°C and *D. buzzatii* was tested from 37°C to 42°C. The stress assays were eventually conducted at 37°C for *D. hydei* and 39.5°C for *D. buzzatii*

**References**

1. Seetharam AS, Stuart GW: **Whole genome phylogeny for 21 Drosophila species using predicted 2b-RAD fragments.** *PeerJ* 2013, **1:**e226.

2. Andrews S: **FastQC: A quality control tool for high throughput sequence data.** *Reference Source* 2010.

3. Marcais G, Kingsford C: **A fast, lock-free approach for efficient parallel counting of occurrences of k-mers.** *Bioinformatics* 2011, **27:**764-770.

4. Bolger AM, Lohse M, Usadel B: **Trimmomatic: a flexible trimmer for Illumina sequence data.** *Bioinformatics* 2014, **30:**2114-2120.

5. Marçais G, Yorke JA, Zimin A: **QuorUM: an error corrector for Illumina reads.** *PLoS One* 2015, **10:**e0130821.

6. Li H: **BFC: correcting Illumina sequencing errors.** *Bioinformatics* 2015, **31:**2885-2887.

7. Zimin AV, Marcais G, Puiu D, Roberts M, Salzberg SL, Yorke JA: **The MaSuRCA genome assembler.** *Bioinformatics* 2013, **29:**2669-2677.

8. Adams MD, Celniker SE, Holt RA, Evans CA, Gocayne JD, Amanatides PG, Scherer SE, Li PW, Hoskins RA, Galle RF, et al: **The genome sequence of *Drosophila melanogaster*.** *Science* 2000, **287:**2185-2195.

9. Boetzer M, Henkel CV, Jansen HJ, Butler D, Pirovano W: **Scaffolding pre-assembled contigs using SSPACE.** *Bioinformatics* 2011, **27:**578-579.

10. Walker BJ, Abeel T, Shea T, Priest M, Abouelliel A, Sakthikumar S, Cuomo CA, Zeng Q, Wortman J, Young SK, Earl AM: **Pilon: an integrated tool for comprehensive microbial variant detection and genome assembly improvement.** *PLoS One* 2014, **9:**e112963.

11. Zaharia M, Bolosky WJ, Curtis K, Fox A, Patterson D, Shenker S, Stoica I, Karp RM, Sittler T: **Faster and more accurate sequence alignment with SNAP.** *arXiv preprint arXiv:11115572* 2011.

12. Simao FA, Waterhouse RM, Ioannidis P, Kriventseva EV, Zdobnov EM: **BUSCO: assessing genome assembly and annotation completeness with single-copy orthologs.** *Bioinformatics* 2015, **31:**3210-3212.

13. Parra G, Bradnam K, Korf I: **CEGMA: a pipeline to accurately annotate core genes in eukaryotic genomes.** *Bioinformatics* 2007, **23:**1061-1067.

14. Waterhouse RM, Zdobnov EM, Tegenfeldt F, Li J, Kriventseva EV: **OrthoDB: the hierarchical catalog of eukaryotic orthologs in 2011.** *Nucleic Acids Res* 2011, **39:**D283-288.

15. Slater GS, Birney E: **Automated generation of heuristics for biological sequence comparison.** *BMC Bioinformatics* 2005, **6:**31.

16. Wu TD, Nacu S: **Fast and SNP-tolerant detection of complex variants and splicing in short reads.** *Bioinformatics* 2010, **26:**873-881.

17. Dobin A, Davis CA, Schlesinger F, Drenkow J, Zaleski C, Jha S, Batut P, Chaisson M, Gingeras TR: **STAR: ultrafast universal RNA-seq aligner.** *Bioinformatics* 2013, **29:**15-21.

18. Pertea M, Pertea GM, Antonescu CM, Chang TC, Mendell JT, Salzberg SL: **StringTie enables improved reconstruction of a transcriptome from RNA-seq reads.** *Nat Biotechnol* 2015, **33:**290-295.

19. Haas BJ, Papanicolaou A, Yassour M, Grabherr M, Blood PD, Bowden J, Couger MB, Eccles D, Li B, Lieber M, et al: **De novo transcript sequence reconstruction from RNA-seq using the Trinity platform for reference generation and analysis.** *Nat Protoc* 2013, **8:**1494-1512.

20. Haas BJ, Delcher AL, Mount SM, Wortman JR, Smith RK, Hannick LI, Maiti R, Ronning CM, Rusch DB, Town CD, et al: **Improving the Arabidopsis genome annotation using maximal transcript alignment assemblies.** *Nucleic Acids Res* 2003, **31:**5654-5666.

21. Lomsadze A, Burns PD, Borodovsky M: **Integration of mapped RNA-Seq reads into automatic training of eukaryotic gene finding algorithm.** *Nucleic Acids Res* 2014, **42:**e119-e119.

22. Majoros WH, Pertea M, Salzberg SL: **TigrScan and GlimmerHMM: two open source ab initio eukaryotic gene-finders.** *Bioinformatics* 2004, **20:**2878-2879.

23. Stanke M, Tzvetkova A, Morgenstern B: **AUGUSTUS at EGASP: using EST, protein and genomic alignments for improved gene prediction in the human genome.** *Genome Biol* 2006, **7:**1-8.

24. Korf I: **Gene finding in novel genomes.** *BMC Bioinformatics* 2004, **5:**59.

25. Haas BJ, Salzberg SL, Zhu W, Pertea M, Allen JE, Orvis J, White O, Buell CR, Wortman JR: **Automated eukaryotic gene structure annotation using EVidenceModeler and the program to assemble spliced alignments.** *Genome Biol* 2008, **9:**R7.

26. Guillen Y, Rius N, Delprat A, Williford A, Muyas F, Puig M, Casillas S, Ramia M, Egea R, Negre B, et al: **Genomics of ecological adaptation in cactophilic *Drosophila*.** *Genome Biol Evol* 2015, **7:**349-366.
