# Supplementary material for: Genomic changes associated with adaptation to arid environments in cactophilic Drosophila species
Source: BMC Genomics. 2019 Jan 16;20:52. doi: 10.1186/s12864-018-5413-3 (PMC6335815; doi:10.1186/s12864-018-5413-3)
Supplement: Supplementary file 2 — Figure S1. Overlap between the orthogroups generating inparalogues in the different species. Figure S2. Overlap between orthogroups belonging to genes under positive selection in each repleta group species. Figure S3. Scheme for heat stress and recovery assay. Table S1. Scaffold and contig length based statistics as well as results from BUSCO genome assessment using the dataset for the five assembled repleta group genomes and D. melanogaster. Table S2. Repeat content analysis for the six species analysed in this study, characterising transposable elements as well as tandem repeats. Table S3. Genome annotation statistics for the five assembled repleta group genomes and D. melanogaster. Table S4. Number of pairwise orthologues between D. melanogaster and the five repleta species, plus the previously published annotation of D. buzzatii. Table S5. Sets of functional terms describing hierarchical grouping of gene ontology terms and the number of genes in Drosophila melanogaster within each set. (DOCX 301 kb) [file 12864_2018_5413_MOESM2_ESM.docx]

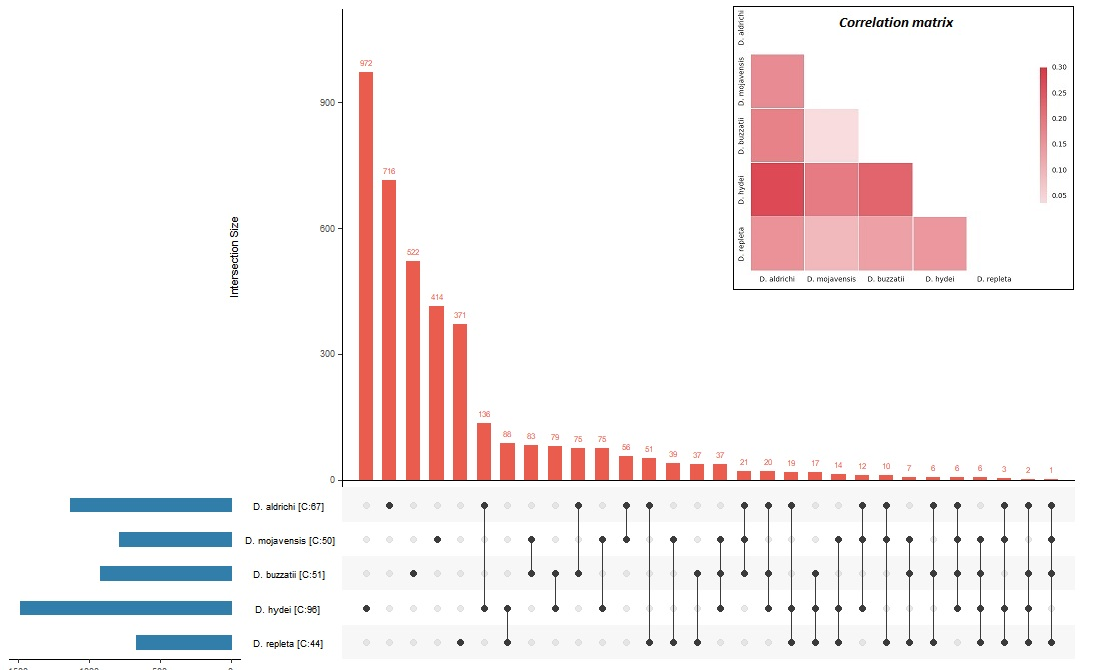


**Figure S1:** Overlap between the orthogroups generating inparalogues in the different species. [C:X] following each species name indicates the number of candidate climatic stress tolerance genes involved. Inset on the top right is the correlation matrix for the orthogroups shared between the different comparisons.


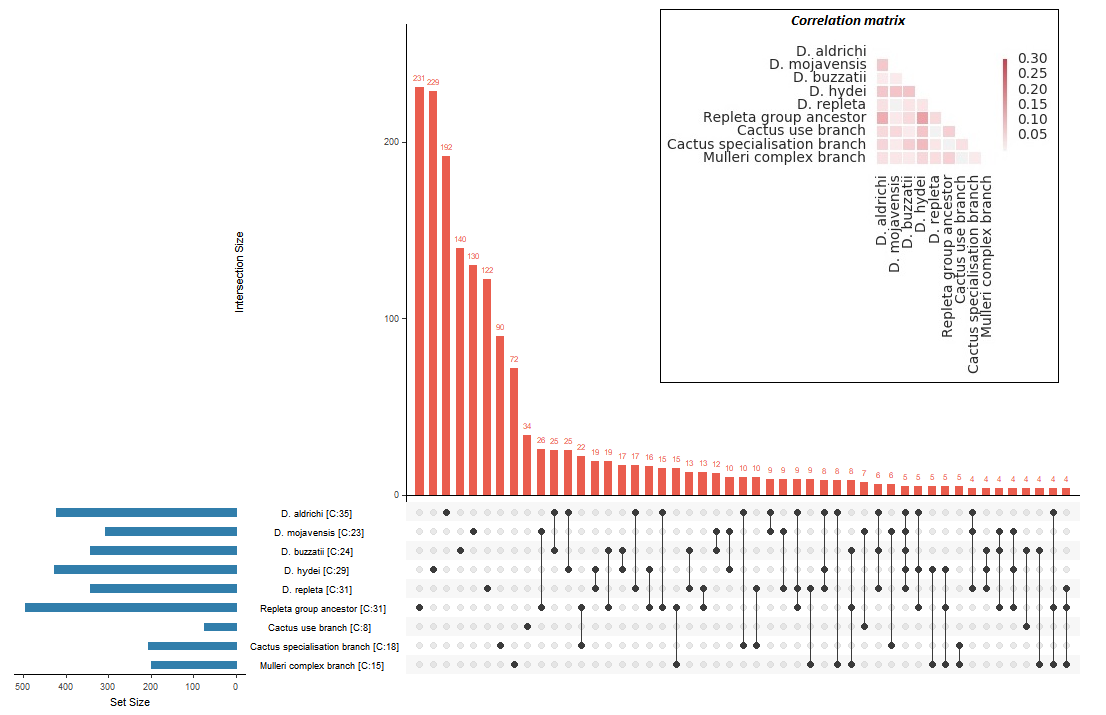


**Figure S2:** Overlap between orthogroups belonging to genes under positive selection in each repleta group species. The [C:X] following the species name indicates the number of candidate climatic stress tolerance genes found within the orthogroups. Inset on the top right is the correlation matrix for orthogroups shared between the different comparisons.


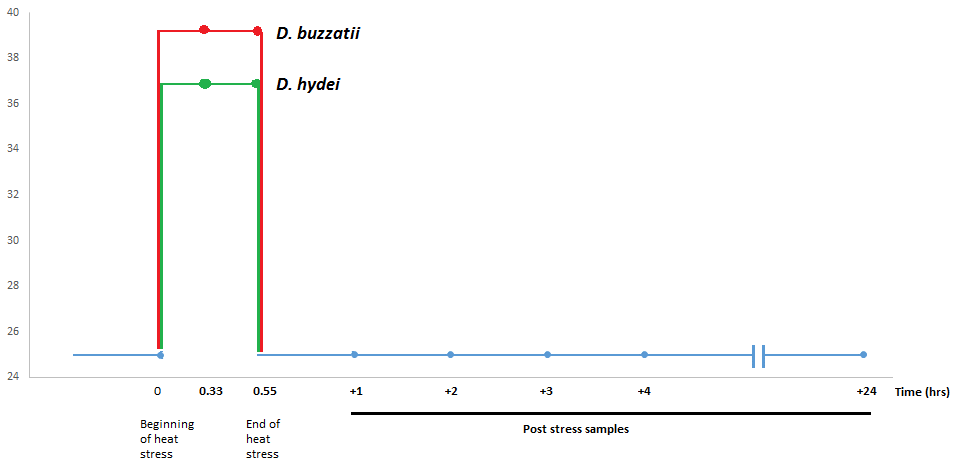


**Figure S3:** Scheme for heat stress and recovery assay. The zero time point was used as a control with two time points during the stress phase and five time points in the recovery phase, including one at 24 hours to assess long-term effects of the stress on recovery. The red line indicates during-stress sampling points for *D. buzzatii* and green indicates the same for *D. hydei*. All blue dots indicate time points sampled for both species. Heat stress was induced via immersion in a heat tank in small vials and recovery took place at 25C

**Table S1:** Scaffold and contig length based statistics as well as results from BUSCO genome assessment using the dataset for the five assembled repleta group genomes and *D. melanogaster.*

| Assembly parameter | *D. aldrichi* | *D. mojavensis* | *D. buzzatii* | *D. hydei* | *D. repleta* | *D. melanogaster* |
| --- | --- | --- | --- | --- | --- | --- |
| Number of scaffolds | 2,620 | 6,841 | 826 | 8,115 | 6,611 | 15 |
| Total size of scaffolds | 190,651,399 | 193,826,310 | 161,490,851 | 165,796,181 | 164,461,345 | 168,736,537 |
| N50 scaffold length | 1,029,703 | 24,764,193 | 1,380,941 | 2,316,993 | 3,476,135 | 23,011,544 |
| L50 scaffold count | 53 | 4 | 30 | 20 | 14 | 4 |
| Gaps in genome (%nucleotides) | 4.48 | 7.03 | 9.27 | 2.22 | 2.09 | 3.77 |
| Average number of contigs per scaffold | 3.4 | 1.7 | 13 | 1.2 | 1.4 | 2506.5 |
| Longest contig (post assembly) | 1,024,094 | 1,453,876 | 448,794 | 4,096,336 | 1,201,580 | 27,905,053 |
| N50 contig length (post assembly) | 83,768 | 124,510 | 28,092 | 468,470 | 207,004 | 19,435,691 |

**Table S2:** Repeat content analysis for the six species analysed in this study, characterising transposable elements as well as tandem repeats.

| Type (as % of genome) | *D. aldrichi* | *D. mojavensis* | *D. buzzatii* | *D. hydei* | *D. repleta* | *D. melanogaster* |
| --- | --- | --- | --- | --- | --- | --- |
| DNA | 11.56 | 3.4 | 4.79 | 5.03 | 9.73 | 1.49 |
| LINE | 4.55 | 2.45 | 1.57 | 2.25 | 4.52 | 4.77 |
| SINE | 0.01 | 0 | 0 | 0.02 | 0.03 | 0 |
| LTR | 7.58 | 6.45 | 1.47 | 4.25 | 8.48 | 11.73 |
| Other | 0.01 | 0 | 0 | 0.01 | 0.01 | 0 |
| Uncharacterised DNA repeats | 0.87 | 2.88 | 0.6 | 0.42 | 0.84 | 0.9 |
| Total | 24.58 | 15.18 | 8.43 | 11.97 | 23.60 | 18.89 |

*DNA – DNA repeats, LINE - long interspersed nuclear elements, SINE – short interspersed nuclear elements, LTR – long terminal repeat

**Table S3:** Genome annotation statistics for the five assembled repleta group genomes and *D. melanogaster.* Statistics to evaluate gene, transcript and exon length as well as alternate splice events were calculated for every genome. (*BUSCO statistics consist of [C]omplete gene models, [D]uplications, [F]ragmented and [M]issing genes).

| Statistic | | *D. aldrichi* | *D. mojavensis* | *D. buzzatii* | | *D. buzzatii (old)* | | *D. hydei* | *D. repleta* | | *D. melanogaster* | | |  |
| --- | --- | --- | --- | --- | --- | --- | --- | --- | --- | --- | --- | --- | --- | --- |
| Mean transcript size(UTR, CDS) | | 2,155 | 2,387 | 2,002 | | 1,636 | | 2,190 | 2,068 | | 2,880 | | |  |
| Mean gene locus size(first to last exon) | | 5,416 | 4,429 | 5,986 | | 3,428 | | 4,982 | 5,016 | | 5,741 | | |  |
| Number of genes | | 16,070 | 14,680 | 14,532 | | 13,158 | | 15,838 | 14,790 | | 13,919 | | |  |
| Number of predicted transcripts | | 20,925 | 20,110 | 17,166 | | 13,158 | | 18,480 | 19,990 | | 30,452 | | |  |
| Mean exon size | | 471 | 439 | 453 | | 397 | | 517 | 478 | | 539 | | |  |
| Number of distinct exons | | 69,409 | 67,288 | 60,185 | | 49,832 | | 67,695 | 61,941 | | 77,694 | | |  |
| Mean number of distinct exons per gene | | 4.3 | 4.3 | 4.1 | | 4.1 | | 4.3 | 4.2 | | 5.58 | | |  |
| Mean number of transcripts per gene | | 1.3 | 1.4 | 1.2 | | 1 | | 1.2 | 1.4 | | 2.19 | | |  |
| Genes with functional annotation | | 13,216 | 12,231 | 11,944 | | 10,496 | | 14,485 | 12,594 | | 12,236 | | |  |
|  | BUSCO complete genes (%) (N = 2,675 genes)* | 95.3 | | | 99.1 | 89 |  | | | 94.8 | | 94.4 | 99 | |
|  | BUSCO duplicate genes (%) | 13 | 9.6 | 12 | |  | | 12 | 9.5 | | 10 | | |  |
|  | BUSCO fragmented genes (%) | 2.2 | 0.8 | 1 | |  | | 2.6 | 3.1 | | 0.7 | | |  |
|  | BUSCO Missing genes (%) | 2.5 | 0.1 | 10 | |  | | 2.6 | 2.5 | | 0.3 | | |  |

**Table S4:** Number of pairwise orthologues between *D. melanogaster* and the five *repleta* species, plus the previously published annotation of *D. buzzatii*.

| Species | ***D. aldrichi*** | ***D. mojavensis*** | ***D. buzzatii*** | ***D. hydei*** | ***D. repleta*** | ***D. melanogaster*** | ***D. buzzatii* (Old annotation)** |
| --- | --- | --- | --- | --- | --- | --- | --- |
| ***D. aldrichi*** | NA | 11880 | 10757 | 11473 | 11334 | 11028 | 10232 |
| ***D. mojavensis*** |  | NA | 11585 | 11539 | 11945 | 11400 | 10480 |
| ***D. buzzatii*** |  |  | NA | 10442 | 10631 | 10314 | NA |
| ***D. hydei*** |  |  |  | NA | 11252 | 10776 | 10412 |
| ***D. repleta*** |  |  |  |  | NA | 10952 | 10405 |
| ***D. melanogaster*** |  |  |  |  |  | NA | 9775 |

**Table S5:** Sets of functional terms describing hierarchical grouping of gene ontology terms and the number of genes in *Drosophila melanogaster* within each set. Each set consists of terms with semantic similarities >0.7 with the major contributing subsets (derived using stricter clustering cut-offs) also listed.

| Set code | Set name | Number of *D. melanogaster* genes | Top subsets |
| --- | --- | --- | --- |
| A | Cell component organization or biogenesis | 2917 | organelle organization; actin filament reorganization involved in cell cycle; cell separation after cytokinesis; cellular component organization or biogenesis; cell projection organization |
| B | Cell fate determination | 31 | single organism reproductive process; developmental cell growth; cell development; cellular developmental process; ectopic germ cell programmed cell death |
| C | Development | 96 | anatomical structure formation involved in morphogenesis; developmental process; single-multicellular organism process; oocyte pole plasm RNA localization; outflow tract morphogenesis |
| D | Cell cycle | 70 | meiotic DNA double-strand break formation; actomyosin contractile ring localization; meiotic cell cycle process; cell cycle process; cell cycle phase transition |
| E | Monooxygenase activity | 568 | superoxide dismutase activity; oxidoreductase activity, acting on the CH-CH group of donors; monooxygenase activity; thioredoxin-disulfide reductase activity; oxidoreductase activity, acting on the CH-NH group of donors |
| F | Hydrolase activity | 353 | lysozyme activity; hydrolase activity, acting on ester bonds; lysophospholipase activity; nuclease activity; ribonuclease activity |
| G | Primary metabolism | 3379 | nitrogen compound metabolic process; primary metabolic process; glycerol ether biosynthetic process; metabolic process; single-organism metabolic process |
| H | Catalytic activity | 3140 | carbon-oxygen lyase activity; N-acetylmuramoyl-L-alanine amidase activity; peptidase activity; hydrolase activity; cyclase activity |
| I | Miscellaneous biological processes | 5177 | biological process; biological adhesion; interspecies interaction between organisms; reproduction; rhythmic process |
| J | Muscle function | 124 | circadian regulation of heart rate; muscle system process; system process; muscle adaptation; renal system process |
| K | Nucleotide sugars metabolism | 144 | chondroitin sulfate metabolic process; nucleotide-sugar metabolic process; amino sugar metabolic process; chitin metabolic process |
| L | Protein localisation | 915 | localization; presynaptic process involved in chemical synaptic transmission; establishment of protein localization; positive regulation of cellular protein localization; establishment of protein localization to mitochondrial membrane |
| M | Energy derivation / oxidation | 174 | molybdopterin cofactor biosynthetic process; nucleotide biosynthetic process; ATP generation from ADP; nucleobase-containing small molecule metabolic process; nucleoside phosphate catabolic process |
| N | Amino acid metabolic process | 207 | cellular amino acid metabolic process; cellular modified amino acid metabolic process; glutamine metabolic process; cellular amino acid biosynthetic process; nitrate assimilation |
| O | Nucleic acid processes | 1529 | meiotic gene conversion; RNA metabolic process; regulation of translational fidelity; cell cycle DNA replication; gene silencing |
| P | Metabolite and ion binding | 4909 | binding; heterocyclic compound binding; nucleotide binding; antigen binding; DNA binding |
| Q | Gene regulation | 494 | mitochondrial RNA metabolic process; regulation of DNA-templated transcription, initiation; regulation of transcription involved in meiotic cell cycle; positive regulation of transcription involved in meiotic cell cycle; chromatin silencing |
| R | Metabolite transport | 896 | renal sodium ion transport; single-organism transport; intracellular transport; cytoplasmic transport, nurse cell to oocyte; nitrogen compound transport |
| S | Homeostasis | 1065 | multicellular organismal iron ion homeostasis; regulation of biological quality; negative regulation of GTPase activity; biological regulation; regulation of catalytic activity |
| T | Receptor regulator activity | 2094 | protein binding; binding, bridging; receptor agonist activity; enzyme binding; transcription factor binding |
| U | Signalling pathways | 541 | stress-activated protein kinase signaling cascade; patched ligand maturation; phototransduction; cellular response to transforming growth factor beta stimulus; regulation of mitochondrial membrane permeability |
| V | Protein modification | 1800 | protein metabolic process; protein methylation; histone methylation; regulation of epidermal growth factor-activated receptor activity; peptidyl-lysine modification to peptidyl-hypusine |
| W | Response to stimulus and stress | 401 | response to chemical; behavioral defense response; response to stress; execution phase of apoptosis; response to stimulus |
